# 14-3-3 scaffold proteins mediate the inactivation of trim25 and inhibition of the type I interferon response by herpesvirus deconjugases

**DOI:** 10.1371/journal.ppat.1008146

**Published:** 2019-11-11

**Authors:** Soham Gupta, Päivi Ylä-Anttila, Tatyana Sandalova, Renhua Sun, Adnane Achour, Maria G. Masucci

**Affiliations:** 1 Department of Cell and Molecular Biology, Karolinska Institutet, Stockholm, Sweden; 2 Science for Life Laboratory, Campus Solna, Stockholm, Sweden; 3 Department of Medicine, Karolinska Institute, Stockholm, Sweden; 4 Division of Infectious Diseases, Karolinska University Hospital, Stockholm, Sweden; University of Southern California, UNITED STATES

## Abstract

The 14-3-3 molecular scaffolds promote type I interferon (IFN) responses by stabilizing the interaction of RIG-I with the TRIM25 ligase. Viruses have evolved unique strategies to halt this cellular response to support their replication and spread. Here, we report that the ubiquitin deconjugase (DUB) encoded in the N-terminus of the Epstein-Barr virus (EBV) large tegument protein BPLF1 harnesses 14-3-3 molecules to promote TRIM25 autoubiquitination and sequestration of the ligase into inactive protein aggregates. Catalytically inactive BPLF1 induced K48-linked autoubiquitination and degradation of TRIM25 while the ligase was mono- or di-ubiquitinated in the presence of the active viral enzyme and formed cytosolic aggregates decorated by the autophagy receptor p62/SQSTM1. Aggregate formation and the inhibition of IFN response were abolished by mutations of solvent exposed residues in helix-2 of BPLF1 that prevented binding to 14-3-3 while preserving both catalytic activity and binding to TRIM25. 14-3-3 interacted with the Coiled-Coil (CC) domain of TRIM25 in *in vitro* pulldown, while BPLF1 interacted with both the CC and B-box domains, suggesting that 14-3-3 positions BPLF1 at the ends of the CC dimer, close to known autoubiquitination sites. Our findings provide a molecular understanding of the mechanism by which a viral deubiquitinase inhibits the IFN response and emphasize the role of 14-3-3 proteins in modulating antiviral defenses.

## Author summary

We have performed a molecular characterization of the mechanism by which the ubiquitin deconjugases encoded in the N-terminal domain of the herpesvirus large tegument proteins inhibit the type I IFN response. Starting from our previous finding that BPLF1, the Epstein-Barr virus (EBV) encoded member of the viral DUB family, induces the formation of a trimolecular complex including TRIM25 and 14-3-3 we now show that the complex promotes both the autoubiquitination and deubiquitination of TRIM25, which leads to sequestration of the ligase into protein aggregates decorated by the autophagy receptor p62/SQSTM1. Using mutants of a conserved putative protein-protein interaction motif in helix-2 of BPLF1 we show that binding to 14-3-3 is essential for this effect and for inhibition of the IFN response. Using 14-3-3 binding mutants in co-immunoprecipitation assays, we found that both BPLF1 and TRIM25 interact with the substrate binding groove of 14-3-3, suggesting that 14-3-3 serves as scaffold for the formation of the trimolecular complex. *In vitro* pulldown assays using TRIM25 subdomains and bacterially expressed BPLF1 and 14-3-3 suggest that 14-3-3 and BPLF1 interact with the tip of the coiled-coil domain, positioning the viral DUB close to a known autoubiquitination site in TRIM25. We used our findings to build a model of the trimeric complex based on available crystal structures and protein docking algorithms. The model provides a first characterization of the molecular interactions involved in the inhibition of TRIM25 by the viral DUB and has interesting implications for the regulation of TRIM25 activity.

## Introduction

The innate immune response is the first line of defense against invading viruses [1]. The response is initiated by the interaction of pathogen-associated molecular patterns (PAMPs) with cellular pattern recognition receptors (PRRs), which triggers intracellular signaling pathways that converge on the activation of a family of canonical and non-canonical inhibitors of nuclear factor kappa-kinases (IKKs) [2]. Activated IKKs promote the phosphorylation and nuclear translocation of transcription factors that regulate the expression of type I interferons (IFN), inflammatory cytokines and other antiviral mediators. The interactions between the components of these signaling pathways are regulated by a variety of post-translational modifications, including the reversible conjugation of ubiquitin (Ub) and ubiquitin-like (UbL) polypeptides, which provides an effective means to control the specificity and magnitude of the response [3].

The covalent attachment of ubiquitin Ub is a three-step process involving enzymes that activate (E1), conjugate (E2) and ligate (E3) the modifier to a Lys residue in the substrate [4]. Ubiquitin itself can be ubiquitinated on different Lys residues, resulting in polyubiquitin chains of different conformation and function [5]. Ubiquitination is reversed by deconjugases (DUBs) that interact with specific substrates and regulate the duration and intensity of signaling [6]. Recent evidence points to a pivotal role of tripartite motif (TRIM) E3 ligases in the regulation of innate antiviral immunity [7, 8]. TRIMs are a family of proteins, comprising over 70 members in humans, that share a molecular organization consisting of an N-terminal really interesting new gene (RING) domain that recognizes the cognate E2, one or two B-boxes (B1/B2) that mediate oligomerization, a coiled-coil (CC) domain that is necessary for dimerization and activation of the ligase, and a variable C-terminal domain that mediates the interaction with specific substrates. The most common C-terminal domain, the PRY-SPRY domain, mediates both protein-protein interactions and binding to RNA [9, 10]. TRIMs control various steps in the innate immune responses including the triggering of PRRs and downstream signaling events leading to the activation of transcription [11]. In addition, TRIMs may directly restrict viral infections by regulating the stability and function of viral proteins that control virus replication and virion assembly [12, 13].

Viruses have evolved multiple strategies to overcome innate immunity to promote their own replication and spread to new hosts [14]. The type I IFN response provides a particularly clear example of the complex interplay between cellular and viral regulators. The TRIM25 ligase activates the responses by ubiquitinating the cytoplasmic viral RNA sensor retinoic acid inducible gene-1 (RIG-I) [15, 16], while the TRIM25-dependent ubiquitination of mitochondrial antiviral signaling proteins (MAVS) terminates signaling [17]. This circuit is controlled by the linear ubiquitination assembly complex (LUBAC) E3 ligase that ubiquitinates the SPRY domain of TRIM25 [18] and by the USP15 deconjugase that regulates the degradation of TRIM25 by the proteasome [19]. Viral proteins target TRIM25 to inhibit RIG-I activation [20]. For example, the non-structural protein 1 (NS1) of influenza A virus displaces the SPRY domain by binding to an overlapping contact site on the CC domain [21], the V proteins of measles, Sendai and parainfluenza viruses bind to the SPRY domain and prevent the interaction of TRIM25 with RIG-I [22], while the E6 proteins of oncogenic HPVs target TRIM25 and USP15 to promote degradation of the ligase [23]. Several members of the herpesvirus family halt the IFN responses by interfering with the ubiquitination and turnover of RIG-I [24, 25]. This may be due to de-ubiquitination of RIG-I by the conserved DUB encoded in the large tegument protein of these viruses. However, the viral enzymes could potentially regulate multiple steps of the signaling cascade and the molecular interactions that lead to the interruption of signaling have not been elucidated. The possible involvement of events upstream of RIG-I ubiquitination is supported by our previous finding that BPLF1, the deconjugase encoded by Epstein-Barr virus (EBV), and the homologs encoded by other herpesviruses, form a trimolecular complex with TRIM25 and members of the 14-3-3 family of molecular scaffold proteins, which activates TRIM25 and promotes autoubiquitination of the ligase [26].

Members of the 14-3-3 family of molecular scaffolds exert an important regulatory function in the IFN response by stabilizing the interaction of TRIM25 with RIG-I and promoting the ubiquitinated-RIG-I-dependent assembly on MAVS filaments, which mediates downstream signaling [27]. The molecular details of this regulatory event are unknown. It is noteworthy that the activity of 14-3-3 proteins is dependent on the formation of homo/heterodimers that interact with phosphorylated motifs in the same or different client protein, which may stabilize active enzyme conformations [28] or promote the formation of protein complexes [29]. The formation of dimers or oligomers, and juxtaposition of RING domains is also required for the activation of TRIM ligases. In some members of the family, stable RING dimers are formed by the packing of hydrophobic residues flanking either side of the core RING domain, whereas in TRIM25 additional interactions, provided by the cognate E2, the substrate or other accessory molecules, are required to stabilize the dimer and activate the ligase [30].

In this study we have sought to elucidate how BPLF1 may halt the type I IFN response by interfering with the activity of the 14-3-3:TRIM25 complex. We found that BPLF1 causes the sequestration of TRIM25 into aggregates that are distinct from the stress granules induced by viral infections and co-localize with the autophagy receptor p62/SQSTM1. Using mutants of known functional domains and protein binding sites in TRIM25, BPLF1 and 14-3-3, we demonstrate that binding of BPLF1 to 14-3-3 is essential for functional incorporation of BPLF1 in the trimolecular complex and inhibition of the IFN response.

## Results

### BPLF1 promotes the autoubiquitination and deubiquitination of TRIM25

**Fig 1:**
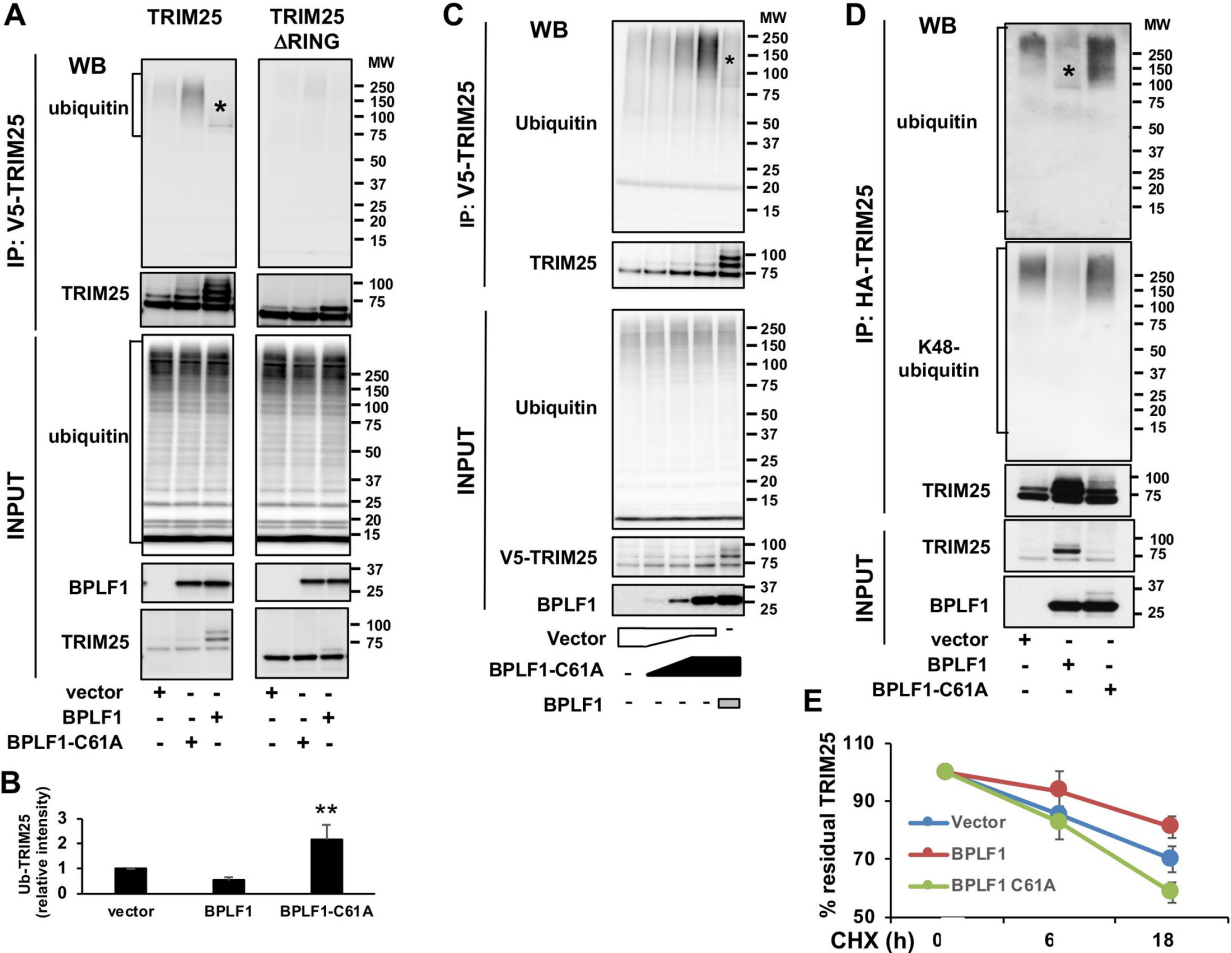
BPLF1 promotes the K48-linked auto-ubiquitination and deubiquitination of TRIM25. HeLa cells were transfected with plasmids encoding V5- or HA-tagged TRIM25 or TRIM25ΔRING and FLAG-tagged catalytically active or inactive BPLF1 and immunoprecipitates of cells harvested 48 h after transfection were probed with the indicated antibodies **A**. Representative western blots illustrating the polyubiquitination of TRIM25 in the presence of catalytic mutant BPLF1, mono-/di-ubiquitination in the presence of the active viral DUB and failure to detect ubiquitinated TRIM25ΔRING. Polyubiquitinated proteins are indicated within brackets and mono-/di-ubiquitinated TRIM25 species are indicated by a star. **B.** Quantification of ubiquitinated TRIM25 in presence of catalytically active and inactive BPLF1 relative to the empty vector. The mean ± SD of four experiments is shown. Statistical analysis was performed using Student's *t*‐test, **P ≤ 0.01. **C.** The effect of catalytically inactive BPLF1 on TRIM25 autoubiquitination is dose dependent while catalytically active BPLF1 can trim the polyubiquitin chains to mono-/di-ubiquitinated species. HeLa cells were co-transfected with V5-TRIM25 and increasing amounts of the FLAG-BPLF1-C61A plasmids. In the last lane catalytically active BPLF1 was added at 1:4 ratio. One representative experiment out of two where western blots of immunoprecipitated V5-TRIM25 were probed with ubiquitin specific antibodies is shown in the figure. **D.** Catalytically inactive BPLF1 promotes the conjugation of K48-linked polyubiquitin chains on TRIM25. HA-TRIM25 was co-transfected with indicated Flag-tagged BLPF1 plasmids and HA-immunoprecipitates were probed with antibodies specific for K48-linked polyubiquitin chains. One representative experiment out of two is shown in the figure. **E.** Cycloheximide chase illustrating the accelerated turnover of endogenous TRIM25 in HeLa cells expressing BPLF1-C61A and stabilization of the ligase by catalytically active BPLF1. The mean ± SD of three experiments is shown.

We have previously reported that the EBV encoded deubiquitinase BPLF1 forms a trimolecular complex with 14-3-3 and the TRIM25 ubiquitin ligase, which leads to autoubiquitination of the ligase and correlates with inhibition of the IFN response [26]. In order to gain insight on the molecular interactions leading to this effect, TRIM25 was immunoprecipitated under denaturing conditions in the presence of cysteine protease inhibitors from HeLa cells co-transfected with V5-tagged TRIM25 or TRIM25ΔRING and the catalytically active (BPLF1) or inactive (BPLF1-C61A) viral DUB. Ubiquitinated TRIM25 was detected in western blots probed with ubiquitin specific antibodies (Fig 1A). The accumulation of high molecular weight polypeptides corresponding to polyubiquitinated TRIM25 was reproducibly observed in cells expressing the catalytically inactive BPLF1-C61A while mono-/di-ubiquitinated TRIM25 accumulated in cells expressing the active viral enzyme (Fig 1A and 1B). TRIM25 ubiquitination was not observed in cells expressing the TRIM25ΔRING mutant suggesting that, while binding of the viral DUB induces autoubiquitination of the ligase, catalytically active BPLF1 may trim the polyubiquitin chains. To test this possibility HeLa cells were co-transfected with plasmids expressing V5-TRIM25 and increasing amounts of FLAG-BPLF1-C61A. The inactive DUB induced a dose-dependent accumulation of polyubiquitinated TRIM25 while addition of a small aliquot of catalytically active BPLF1 resulted in the accumulation of mono/di-ubiquitinated species (Fig 1C). A similar BPLF1-dependent autoubiquitination and deubiquitination was observed for endogenous TRIM25, confirming that the effect is not an artifact of overexpression (S1 Fig). Probing the TRIM25 immunoprecipitates with antibodies specific for different types of ubiquitin isopeptide linkages revealed that BPLF1-C61A promotes the formation of K48-linked polyubiquitin chains (Fig 1D), suggesting that the inactive DUB may induce degradation of the ligase. This was confirmed in cycloheximide chase experiments where the degradation of TRIM25 was enhanced in cells expressing BPLF1-C61A while catalytically active BPLF1 stabilized the ligase (Fig 1E).

### BPLF1 induces the formation of TRIM25 aggregates

**Fig 2:**
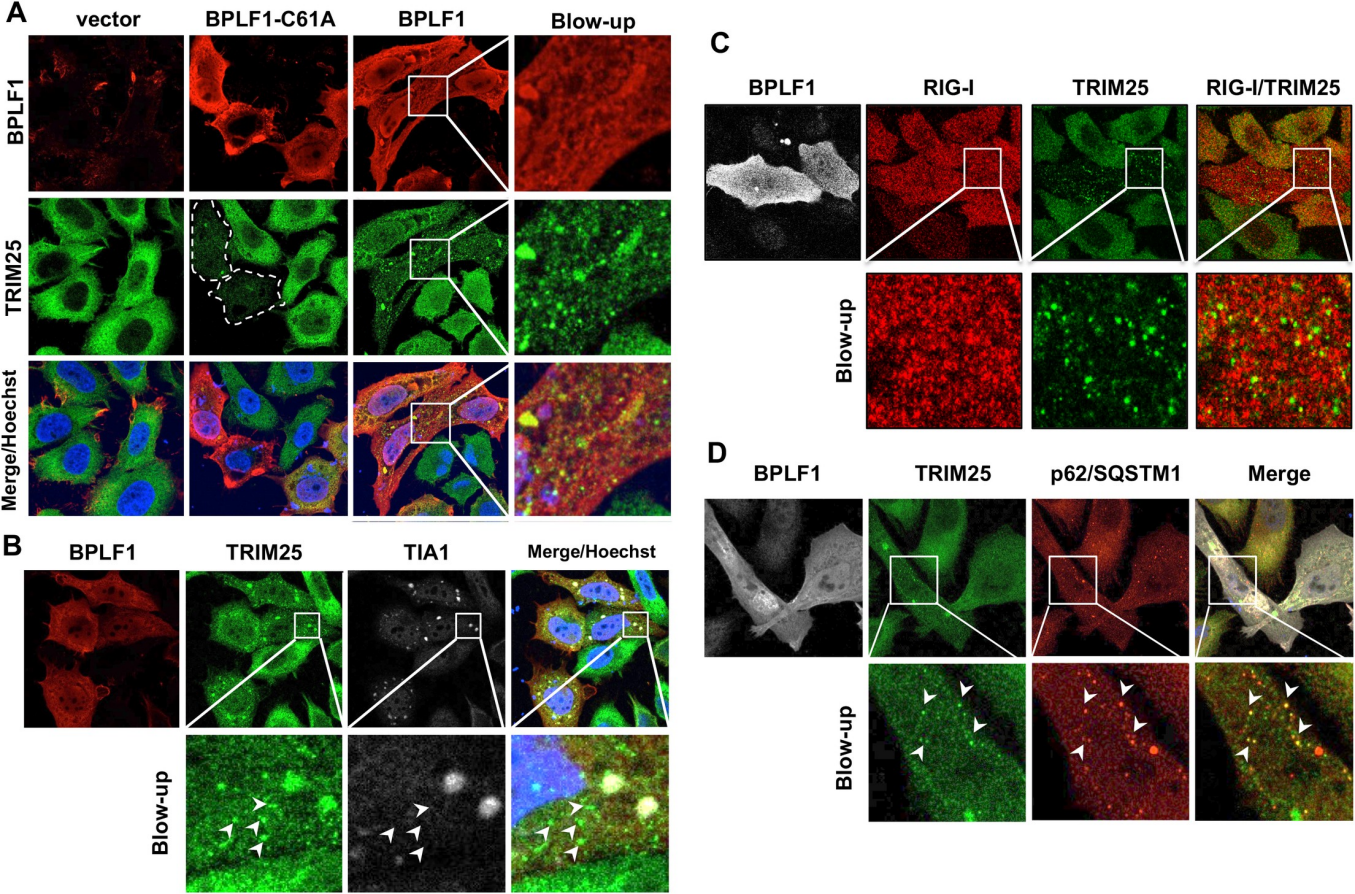
Catalytically active BPLF1 promotes the formation of TRIM25 aggregates. HeLa cells transfected with FLAG-tagged catalytically active (BPLF1) or inactive (BPLF1-C61A) BPLF1 were stained 24 h after transfection with the indicated specific antibodies. **A.** Representative micrographs illustrating the formation of TRIM25 aggregates in cells expressing active BPLF1 and the decrease of TRIM25 fluorescence in cells expressing the inactive enzyme. Confocal images were obtained at 63x lens objective magnification. Endogenous TRIM25 is in green, BPLF1 is in red and cell nuclei were stained with Hoechst (blue). **B**. Representative micrographs illustrating the failure of BPLF1 induced TRIM25 aggregates to co-localize with the RNA binding protein T cell intracellular antigen (TIA)-1, a bona fide marker of stress granules. Only large TRIM25 aggregates colocalize with TIA-1 while the majority of small aggregates, indicated by white arrows, are TIA-1 negative. **C**. Representative micrographs illustrating the failure of RIG-I to accumulate in the TRIM25 aggregates induced by wild type BPLF1. Endogenous RIG-I (red) and TRIM25 (green) were co-stained in HeLa cells transfected with wild type BPLF1 (gray) **D.** Representative micrographs illustrating the co-localization of TRIM25 aggregates with the ubiquitin-dependent autophagy receptor p62/SQSTM1. Endogenous TRIM25 (green) and p62/SQSTM1 (red) were co-stained in HeLa cells transfected with wild type BPLF1 (gray).

Activation of the antiviral response is accompanied by the relocalization of effector molecules into subcellular compartments where signaling is initiated and amplified [31]. Upon activation of the IFN response TRIM25 and RIG-I colocalize into stress granules that are critical for signaling [31, 32]. In order to investigate whether the expression of BPLF1 is sufficient to alter the subcellular localization of TRIM25 in the absence of IFN triggering, TRIM25 was visualized by immunofluorescence in HeLa cells expressing catalytically active or inactive BPLF1. As illustrated by the confocal images shown in Fig 2A, TRIM25 exhibited a diffuse mostly cytoplasmic fluorescence in vector transfected cells. Large TRIM25 aggregates were occasionally observed in the transfected cells independently of BPLF1 expression. In line with the induction of K48-linked polyubiquitination and enhanced turnover, cells expressing the catalytic mutant BLPF1-C61A showed an overall decrease of TRIM25 fluorescence, which appeared to be stronger in cells expressing higher levels of the viral protein (Figs 2A and S2A). In contrast, expression of the active viral DUB was associated with a remarkable reorganization of TRIM25 fluorescence into small to medium size aggregates that appeared distinct from large aggregates induced by transfection. Scoring for different patterns of TRIM25 fluorescence in repeated experiments confirmed the formation of TRIM25 aggregates in the majority of cells expressing catalytically active BPLF1 (S2B Fig) and significant decrease of TRIM25 fluorescence in cells expressing the BPLF1-C61A mutant (S2C Fig). Staining with antibodies specific for the RNA binding protein TIA-1, a bona fide marker of stress granules that are induced by viral infection [33, 34], revealed that the vast majority of the small/medium size aggregates were TIA-1 negative, whereas TRIM25 co-localized with TIA-1 in the large aggregates that were observed also in vector transfected cells (Fig 2B). Thus, the aggregates induced by BPLF1 do not appear to be authentic stress granules. In line with their formation in the absence of IFN triggering, the majority of the aggregates did not colocalize with RIG-I (Fig 2C). Co-staining with antibodies specific for different types of cytoplasmic bodies revealed co-localization of the TRIM25 aggregates with the ubiquitin binding sequestosome protein p62/SQSTM1 (Fig 2D) that targets ubiquitinated cargo for degradation by autophagy.

**Fig 3:**
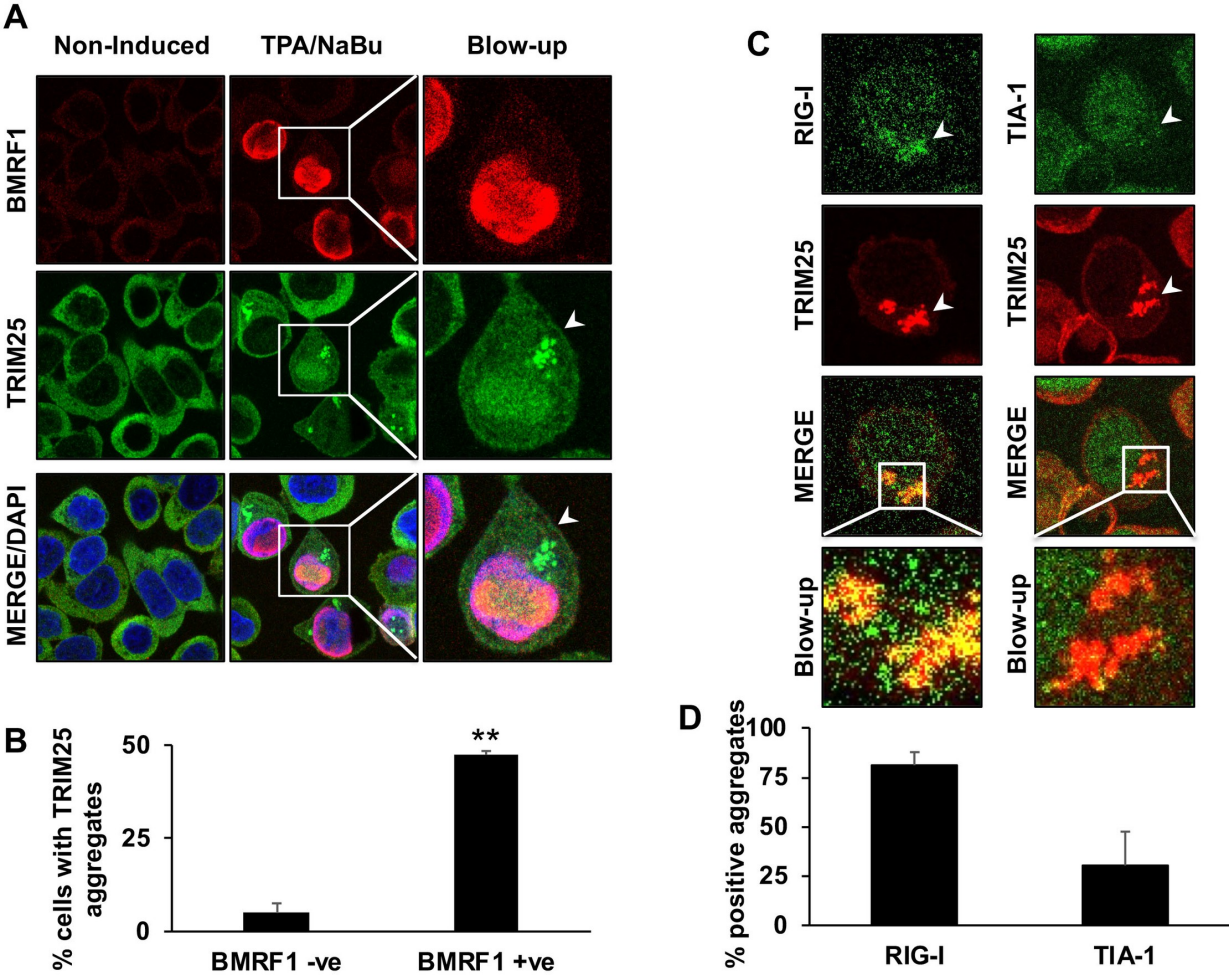
TRIM25 aggregates are induced upon reactivation of the productive cycle in EBV positive cells. The productive virus cycle was induced in AGS-Bx1 cells by treatment with TPA/NaBu and induced cells were visualized by staining with antibodies specific for the early antigen BMRF1 (red). **A**. Representative micrographs illustrating the formation of TRIM25 aggregates (green) in BMRF1 positive cells. **B.** Quantification of the number of BMRF1 positive and negative cells exhibiting TRIM25 aggregates. The mean ± SD of two independent experiments is shown. Statistical analysis was performed using Student's *t*‐test, **P ≤ 0.01. **C.** Representative micrographs illustrating the co-localization of RIG-I with TRIM25 aggregates and failure of the aggregates to co-localize with TIA-1. **D.** Quantification of the number of TRIM25 aggregates co-stained with RIG-I and TIA-1 specific antibodies. The mean ± SD of two independent experiments is shown.

In order to assess whether TRIM25 aggregates may form also upon physiological levels of BPLF1 expression, the productive virus cycle was induced in EBV positive AGS-Bx1 cells by treatment with TPA and sodium butyrate (TPA/NaBu) and TRIM25 was visualized by immunofluorescence while virus reactivation was confirmed by staining for the early antigen BMRF1. As illustrated by the representative micrograph shown in Fig 3A, and quantification of aggregate positive cells (Fig 3B), TRIM25 aggregates were readily detected in the majority of productively infected cells but only rarely observed in BMRF1 negative cells. In line with the triggering of IFN response during productive infection, the majority of the aggregates colocalized with activated RIG-I, while they were mostly negative for TIA-1 (Fig 3C and 3D).

### Binding to 14-3-3 is required for the formation of TRIM25 aggregates and for inhibition of the IFN response

**Fig 4:**
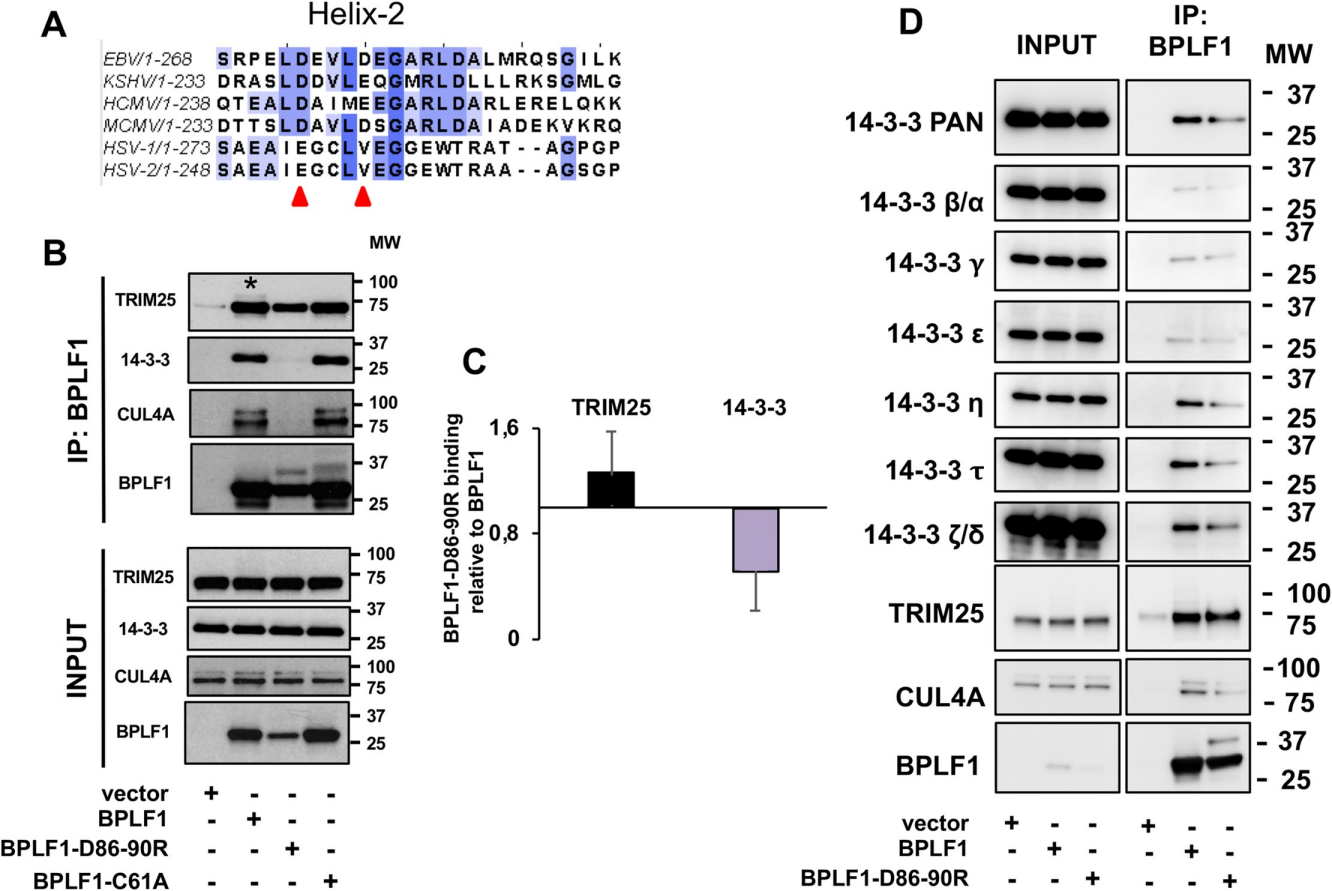
Helix-2 of the viral DUBs is involved in binding to 14-3-3. **A**. Clustal-omega generated multiple sequence alignment of the conserved helix-2 of herpesvirus deconjugases. Solvent exposed acidic residues that were mutated in BPLF1 are indicated by red arrows. **B**. The mutation in helix-2 selectively impairs the binding of BPLF1 to 14-3-3. HeLa cells were transfected with the indicated BPLF1 Flag-tagged plasmids and cell lysates were immunoprecipitated with anti-FLAG conjugated agarose beads (IP). Western blots were probed with the indicated antibodies. One representative experiment out of three where the wildtype and mutant BPLF1 were tested in parallel is shown in the figure. **C**. The intensity of the TRIM25 and 14-3-3 specific bands were quantified by densitometry and relative binding was calculated as the ratio between the intensity of the BPLF1-D86-90R and BPLF1 immunoprecipitates. The means ± SD of three experiments are shown. **D.** Mutation of helix-2 inhibits the binding of BPLF1 to all 14-3-3 isoforms. BPLF1 and BPLF1-D86-90R mutant were immunoprecipitated from transfected Hela cells and western blots were probed with 14-3-3 isoform specific antibodies.

In addition to the conserved Cis-box and His-box that form the catalytic core of the enzymes, the solvent exposed helix-2 is relatively well conserved amongst the herpesvirus encoded homologs and we have previously shown that this domain mediates the binding of BPLF1 to cullin ligases [35]. In order to test whether helix-2 mediates the interaction between BPLF1 and the TRIM25:14-3-3 complex, we took advantage of a previously described BPLF1 mutant where the solvent exposed Asp residues in position D86 and D90 were substituted with Arg [35] (Fig 4A). The mutant retains catalytic function as confirmed by labeling with the Ub-VS and Ub-VME functional probes (S3A Fig). HeLa cells were transfected with FLAG-tagged BPLF1, BPLF1-D86-90R, BPLF1-C61A and empty FLAG-vector, and FLAG immunoprecipitates were probed with antibodies to TRIM25 and 14-3-3. Although the expression of BPLF1-D86-90R mutant was somewhat lower compared to wild-type and catalytic mutant BPLF1 (Fig 4B), the relative binding to TRIM25 was not significantly affected while binding to 14-3-3 was severely impaired. A similar impairment was observed when the BPLF1 and BPLF1-D86-90R immunoprecipitates were probed with antibodies specific for several 14-3-3 isoforms (Fig 4D), suggesting that helix-2 of BPLF1 engages a conserved domain in the molecular scaffolds.

**Fig 5:**
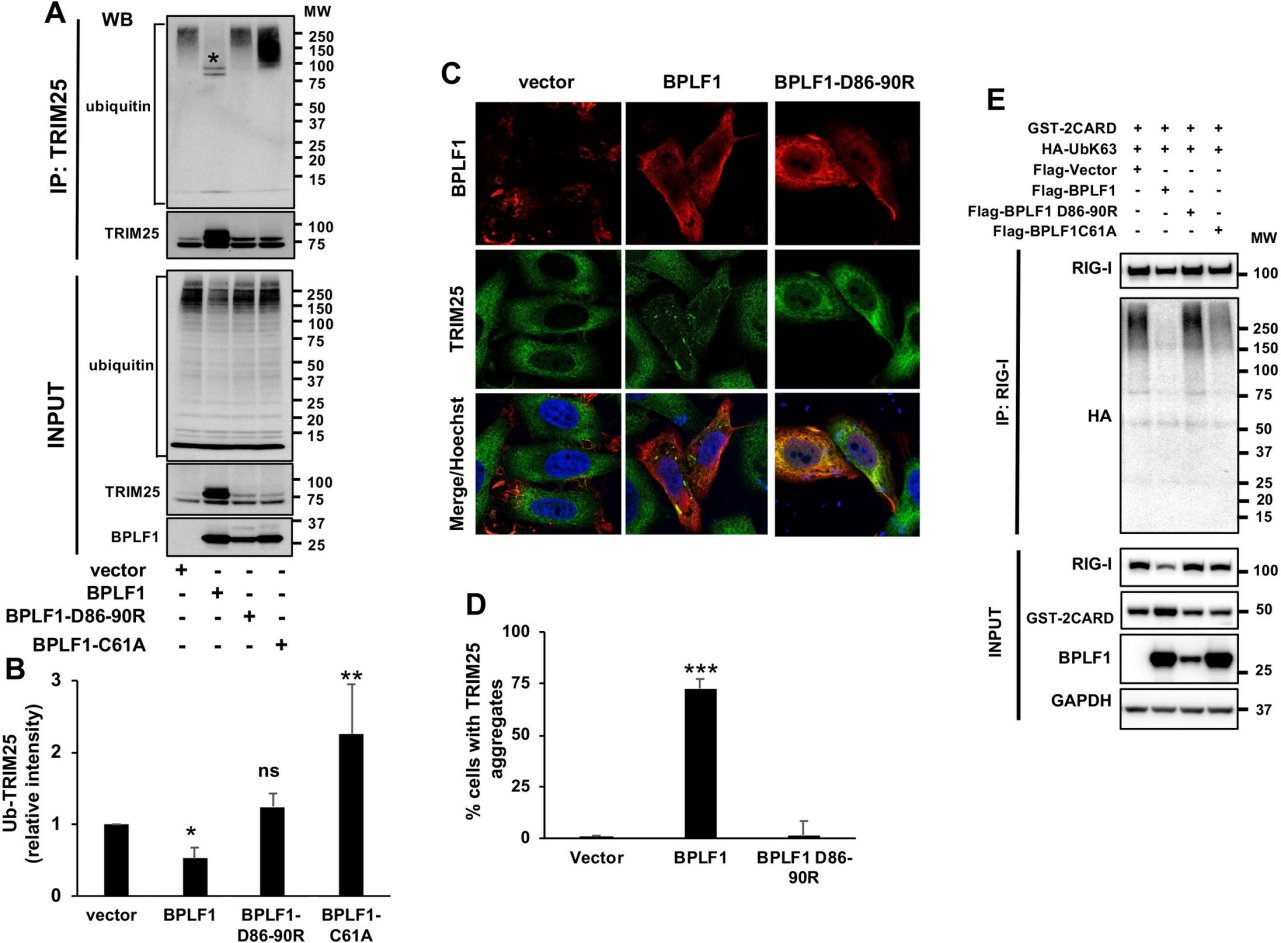
Binding of BPLF1 to 14-3-3 is required for TRIM25 autoubiquitination and aggregate formation. **A.** BPLF1-D86-90R fails to induce TRIM25 autoubiquitination. HA-TRIM25 was co-transfected in Hela cells together with indicated Flag-tagged plasmids. HA-immunoprecipitates were probed with a polyubiquitin specific antibody. **B.** The intensity of ubiquitinated TRIM25 was quantified by densitometry. The mean ± SD of three experiments is shown. Statistical analysis of the difference between vector and BPLF1 expressing cells was performed using Student's *t*‐test: ns = P >0.05, * P ≤0.*05*, ** P ≤0.01. **C.** BPLF1-D86-90R does not induce the formation of TRIM25 aggregates. Confocal images were obtained at 63x lens objective magnification. TRIM25 is in green, BPLF1 is in red and cell nuclei were stained with Hoechst (blue). **D.** Quantification of the percentage of BPLF1 positive cells exhibiting TRIM25 aggregates. Cells showing clear redistribution of TRIM25 in small aggregates were scored as positive. The mean ± SD of two independent experiments is shown. Statistical analysis was performed using Student's *t*‐test, ***P ≤0.001. **E.** BPLF1-D86-90R fails to inhibit the ubiquitination of endogenous RIG-I upon triggering of the IFN response. HA-UbK63 was co-transfected in HeLa cells together with the indicated FLAG-tagged plasmid. Activation of the IFNβ pathway was induced by co-transfection of GST-2CARD. Endogenous RIG-I was immunoprecipitated and ubiquitination was detected by probing immunoblots with the anti-HA antibody.

**Fig 6:**
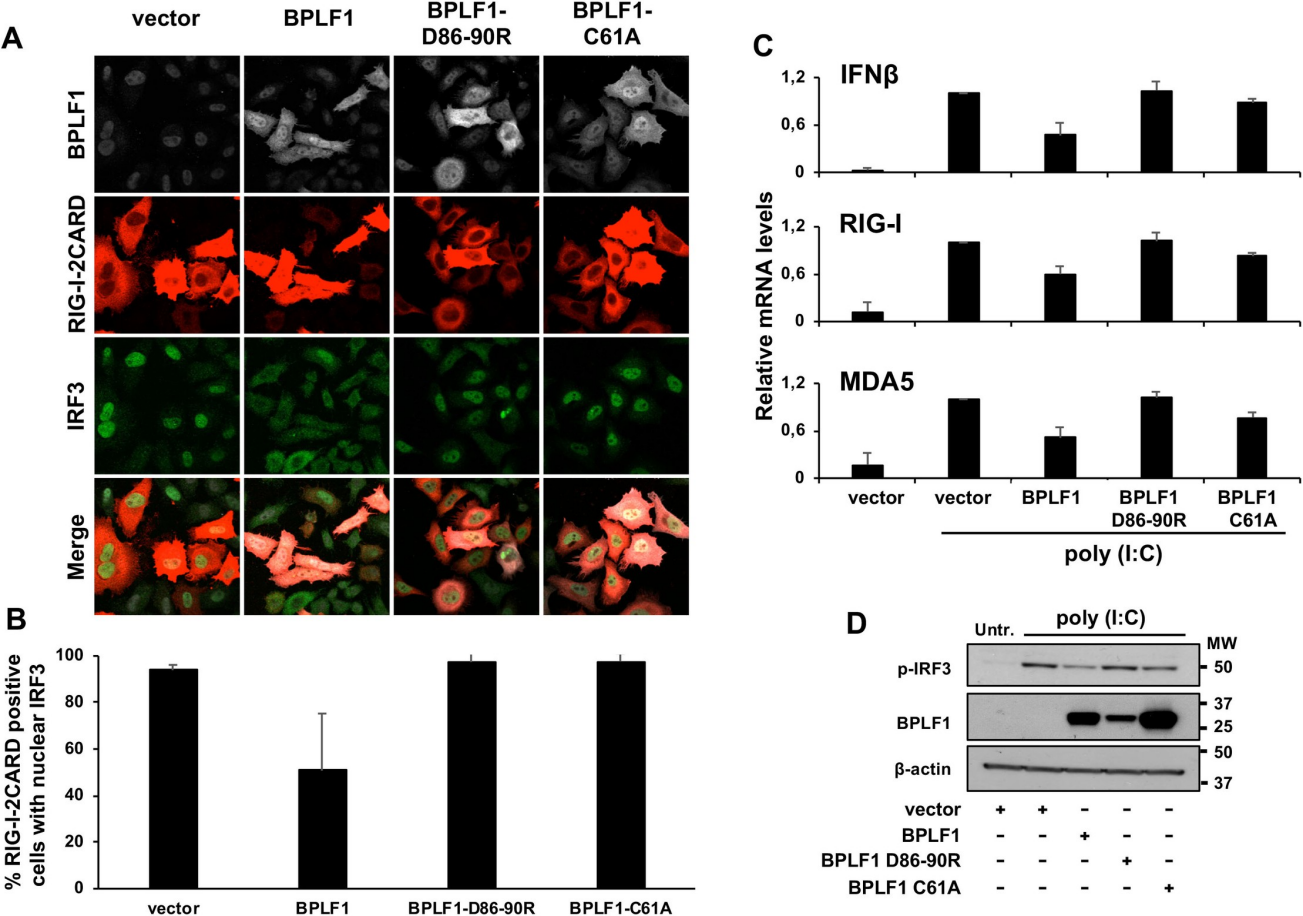
Binding of BPLF1 to 14-3-3 is required for inhibition of the IFN response. **A**. The BPLF1-D86-90R fails to inhibit the nuclear translocation of IRF3 in cells expressing constitutively active RIG-I-2CARD. Confocal images were obtained at 63x lens objective magnification. IRF3 is in green, RIG-I-2CARD in red, BPLF1 in grey. **B**. Quantification of the percentage of cells exhibiting nuclear IRF3. The mean ± SD of two independent experiments is shown. **C.** BPLF1-D86-90R fails to inhibit the transcription of IFN and IFN-regulated genes induced by treatment with poly(I:C). The levels of specific mRNAs were quantified by qRT-PCR. The results are shown as fold change relative to vector transfected poly(I:C) treated cells. The mean ± SD of two experiments each performed in triplicate is shown. **D**. Representative western blots illustrating the expression of phosphorylated IRF3 in the experiment shown in Fig 6C. Phospho-IRF3 is downregulated in cells expressing catalytically active BPLF1 but not in cells expressing BPLF1-D86-90R. One representative experiment out of two is shown in the figure.

We then tested whether the failure of BPLF1-D86-90R to interact with 14-3-3 affects its capacity to inhibit the type I IFN response. Expression of the mutant did not enhance the accumulation of polyubiquitinated TRIM25 or mono-/di-ubiquitinated species in transfected HeLa cells (Fig 5A and 5B), even when the relatively lower expression of the mutant was compensated by transfecting a 12-fold higher amount of the plasmid (S3B Fig). Furthermore, the BPLF1-D86-90R mutant was unable to induce the formation of TRIM25 aggregates (Fig 5C and 5D) and did not prevent the ubiquitination of RIG-I upon triggering of the IFN response by transfection of the constitutively active RIG-I-2CARD (Fig 5E). In line with these findings, the mutant failed to inhibit the IFN response as illustrated by preserved nuclear translocation of phosphorylated IRF3 in cells expressing the constitutively active RIG-I-2CARD (Fig 6A and 6B), transcriptional activation of IFNβ and the IFNβ regulated genes RIG-I and MDA5 (Fig 6C) and accumulation of phosphorylated IRF3 in HeLa cells treated with poly(I:C) (Fig 6D). Collectively, these findings support the conclusion that binding of BPLF1 to 14-3-3 is critical for its recruitment to the 14-3-3:TRIM25 complex and for the capacity of the viral DUB to inhibit the IFN response.

### Molecular interactions in the BPLF1:14-3-3:TRIM25 complex

**Fig 7:**
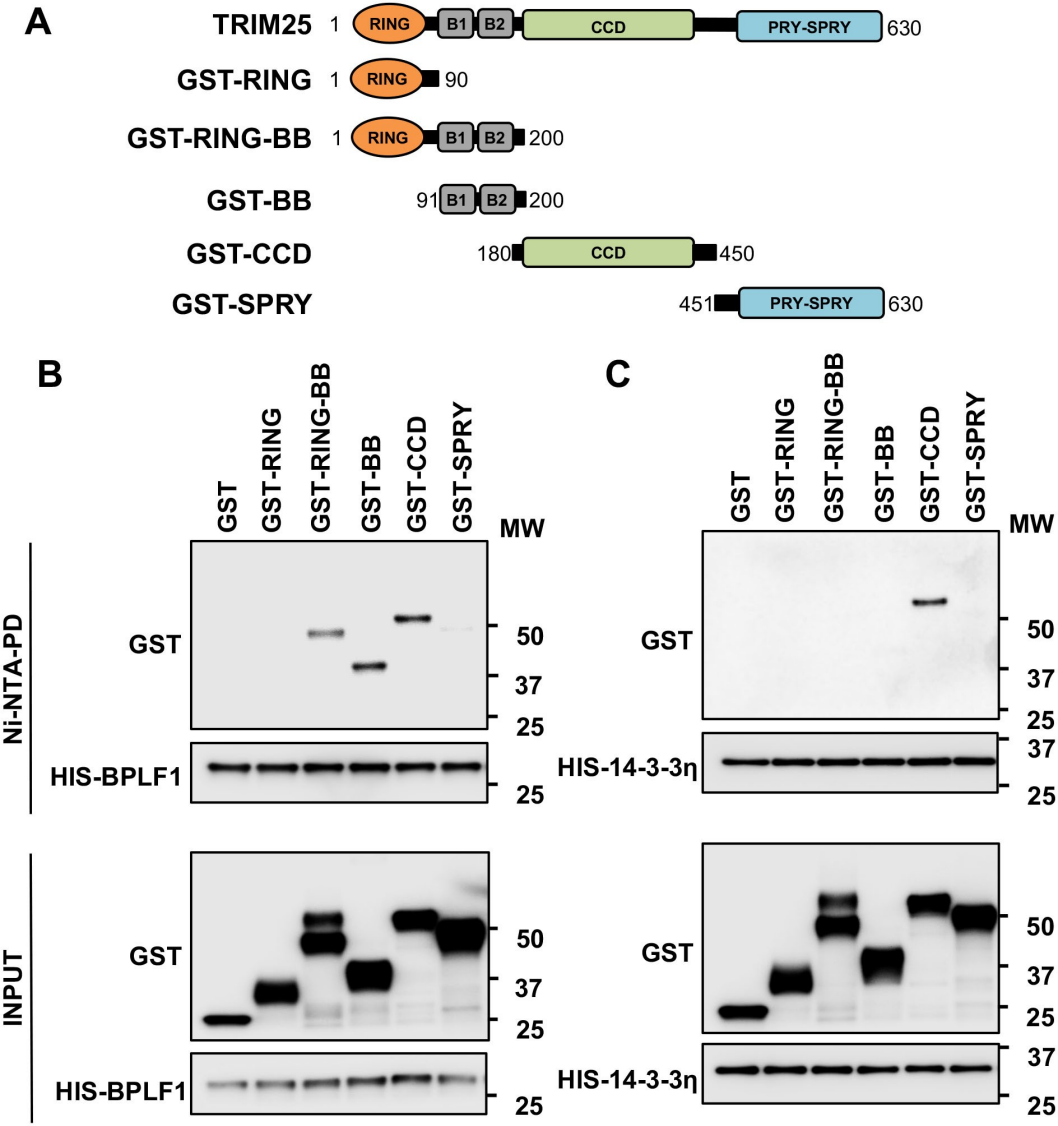
14-3-3 and BPLF1 interact with the tip of the TRIM25 CC dimer. **A.** Schematic illustration of the domain structure and amino acid coordinates of TRIM25 and the GST-tagged constructs. The TRIM25 domains are color coded: N-terminal RING (RING, orange), B-box domain (B1-B2, grey), Coiled-Coil domain (CCD, lime), PRY-SPRY domain (SPRY, blue). **B.** *In vitro* binding assay illustrating the interaction of BPLF1 with the B-box and CCD domains of TRIM25. Comparable amounts of purified His-BPLF1 and GST-TRIM25 domains were mixed and His-BPLF1 was purified with Ni-NTA beads. Immunoblots were probed with anti-His and anti-GST-antibodies. Input corresponds to 10% of the volume used for pulldown. One representative experiment out of two is shown in the figure. **C.** *In vitro* binding assay illustrating the interaction of 14-3-3 with the CC domain of TRIM25. Comparable amounts of bacterially expressed His-14-3-3η and immunoprecipitated GST-TRIM25 domains were mixed and His-14-3-3η was captured with Ni-NTA beads. Immunoblots were probed with anti-His and anti-GST-antibodies. Input corresponds to 10% of the volume used for pulldown. One representative experiment out of two is shown in the figure.

We then sought to characterize the molecular interactions involved in the recruitment of BPLF1 to the 14-3-3:TRIM25 complex. To this end we took advantage of a set of TRIM25 deletion mutants expressing the N-terminal RING domain (GST-RING), the B-box1 and -2 (GST-BB), the RING and B-box domains (GST-RING-BB), the coiled-coil domain (GST-CCD), and the C-terminal PRY-SPRY domain (GST-SPRY) fused to glutathione-*S*-transferase (GST) (Fig 7A). The GST-tagged polypeptides were immunoprecipitated from transfected HeLa cells under denaturing conditions to prevent contamination by other cellular proteins and equal amounts of the precipitates were mixed with bacterially expressed His-tagged BPLF1 or 14-3-3η at approximately equimolar ratios. Since the capacity of BPLF1 to interact with all 14-3-3 isoform suggests the involvement of a conserved domain, 14-3-3η was chosen for these experiments based on the relatively stronger interaction with BPLF1 in co-immunoprecipitation experiments (Fig 4D) and reported constitutive interaction with TRIM25 [27], making this isoform more suitable for *in vitro* binding studies. Probing the His pulldowns with GST specific antibodies revealed that BPLF1 interacts with RING-BB, BB and CCD fragments of TRIM25 (Fig 7B) while 14-3-3 interacts only with the CC domain (Fig 7C).

**Fig 8:**
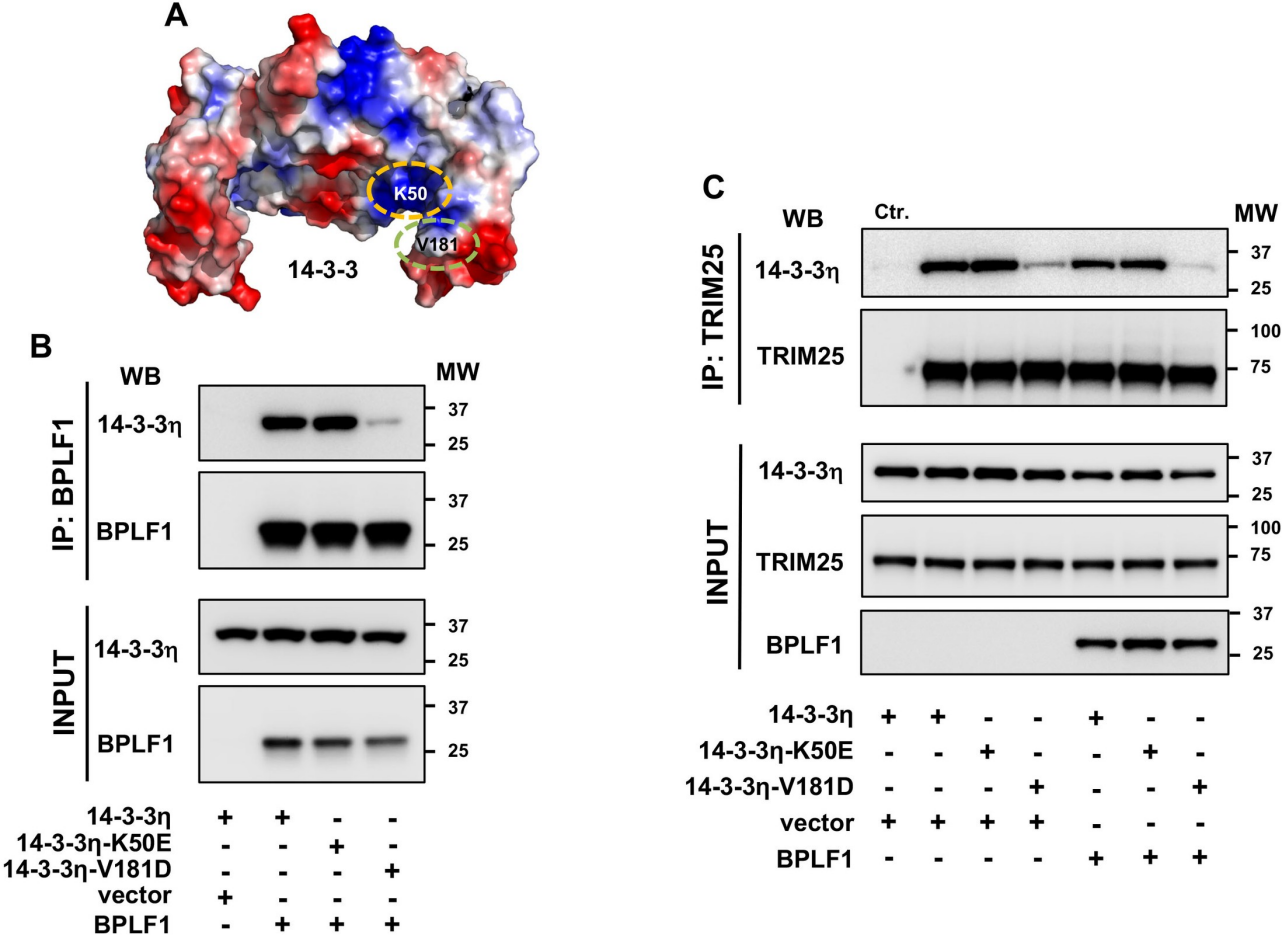
BPLF1 and TRIM25 interact with the substrate binding groove of 14-3-3. **A.** Surface electrostatic potential of the crystal structure of the 14-3-3η homodimer. Charged surfaces are displayed in shades of blue (positive), red (negative) and white (non-polar). The positions of the K50E and V181D mutations are indicated. **B.** BPLF1 binds to 14-3-3η and 14-3-3η-K50E but not to 14-3-3n-V180D. HeLa cells were co transfected with Flag-BPLF1 and the indicated Myc-tagged 14-3-3η constructs and BPLF1 immunoprecipitates were probed with antibodies to the Myc and FLAG tags. One representative experiment out of two is shown in the figure. **C.** TRIM25 interacts with 14-3-3η, 14-3-3η-K50E but not with 14-3-3η-V181D and the binding is not affected by co-expression of BPLF1. HeLa cells were co-transfected with Flag-BPLF1 and the indicated Myc-tagged 14-3-3η constructs. Endogenous TRIM25 was immunoprecipitated (IP) and immunoblotted (WB) with c-Myc antibody. One representative experiment out of two is shown in the figure. Ctr. = Mock-IP was performed with an isotype matched irrelevant antibody.

Members of the 14-3-3 protein family form homo/heterodimers that adopt a horse-shoe like conformation with a highly conserved inner surface and a variable outer surface. The inner surface encloses an amphipathic groove that features a positively charged crevice at one end and a hydrophobic patch at the other (Fig 8A). The positively charged crevice contains a conserved Lys (14-3-3η-K50) and a triad of Arg (14-3-3η-R57, -R61 and -R132) residues that mediate binding to phosphorylated peptides/proteins while both phosphorylated or unphosphorylated substrates contact a hydrophobic patch that in 14-3-3η includes the V181 residue [36]. In order to map the interaction of 14-3-3 with TRIM25 and BPLF1 we used two binding mutants of 14-3-3η where K50 and V181 were mutated to Glu and Asp, respectively [37–39]. Immunoprecipitation of BPLF1 from HeLa cells co-transfected with wild type or mutant 14-3-3η revealed that the K50E mutation did not affect the interaction with BPLF1, while binding was virtually abolished by the V181D mutation (Fig 8B). A similar pattern of interaction was observed when TRIM25 was immunoprecipitated from HeLa cells transfected with wild type or mutant 14-3-3η in the presence or absence of BPLF1 (Fig 8C), suggesting that both proteins contact the conserved amphipathic groove of 14-3-3, which is in line with the capacity of both BPLF1 and TRIM25 to interact with several 14-3-3 isoforms. The capacity of the 14-3-3η-K50E mutant to interact with both client proteins suggest that binding is not dependent on phosphorylation, which was confirmed by the similar efficiency of co-immunoprecipitation independently of addition of phosphatase inhibitors to the lysis buffer (S4A and S4B Fig).

**Fig 9:**
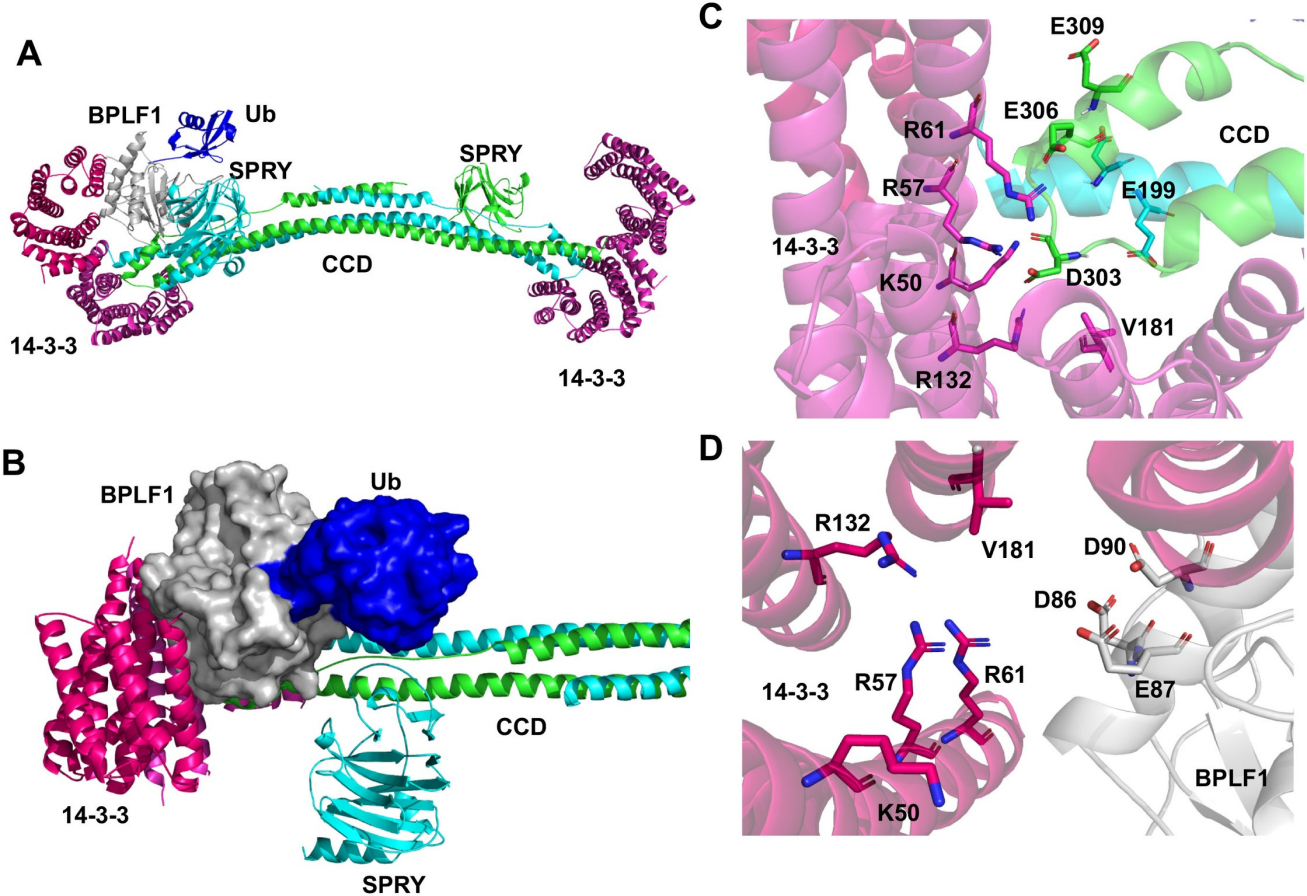
Predicted mode of interaction of 14-3-3 with BPLF1 and TRIM25. The results of *in vitro* binding assays and available crystal structures of the three binding partners were used to build a model of the BPLF1:14-3-3:TRIM25 complex. High probability molecular interactions were predicted using the ClusPro server. **A.** Side view of the complex of the TRIM25 CCD-SPRY dimer (green and cyan) with 14-3-3 (purple) and BPLF1 (grey) bound to ubiquitin (blue) illustrating how engagement of the two binding sites in the groove of the 14-3-3 dimer could position BPLF1 close to the tip of the CCD domain of TRIM25. **B.** Top view of the same complex with space filled models of BPLF1 and ubiquitin. **C.** Close-up view of the predicted interaction of 14-3-3 (pink) with the CC domain of the TRIM25 dimer (green and cyan). **D.** Close-up view of the predicted interaction of BPLF1 helix-2 (grey) with the binding groove of 14-3-3 (pink).

In order to gain insight on the architecture of the complex and the molecular interaction that promote the activation and inactivation of TRIM25, the ClusPro server [40] was used to predict protein-protein docking based on the crystal structures of the TRIM25 CCD-SPRY domain [21] and 14-3-3 in open conformation [41], and on a homology model of BPLF1 based on the crystal structure of the murine cytomegalovirus M48 homolog [42]. The highest scoring model that could accommodate the *in vitro* binding data was selected for analysis. Side and top views of the predicted site of interaction between 14-3-3, BPLF1 and the CC domain of TRIM25, and blow up of the docking of BPLF1 and TRIM25 in the binding groove of 14-3-3 are shown in Fig 9. The model predicts that the binding pocket of 14-3-3 could simultaneously engage the tip of the CC domain of TRIM25 and helix-2 in BPLF1, positioning the catalytic site of BPLF1 facing towards the center of the TRIM25 dimer (Fig 9A and 9B). The acidic residues of helix-2 direct BPLF1 towards the crevice of the 14-3-3 binding groove forming strong interactions with the cluster of positively charged residues K50, R57, R61 and R132 (Fig 9C). The double D86-90R mutation in helix-2 may prevent binding through electrostatic repulsion while the presence of several positively charged residues may attenuate the effect of the 14-3-3-K50E mutation. Binding would also be inhibited by the 14-3-3-V181D mutation that creates an electrostatic clash with the acidic surface of helix-2. A similar architecture and effect of the K50E and V181D mutations are predicted for the interaction of 14-3-3 with the acidic residues D303, E306 and E309 on the tip of the CC domain of TRIM25 (Fig 9D).

**Fig 10:**
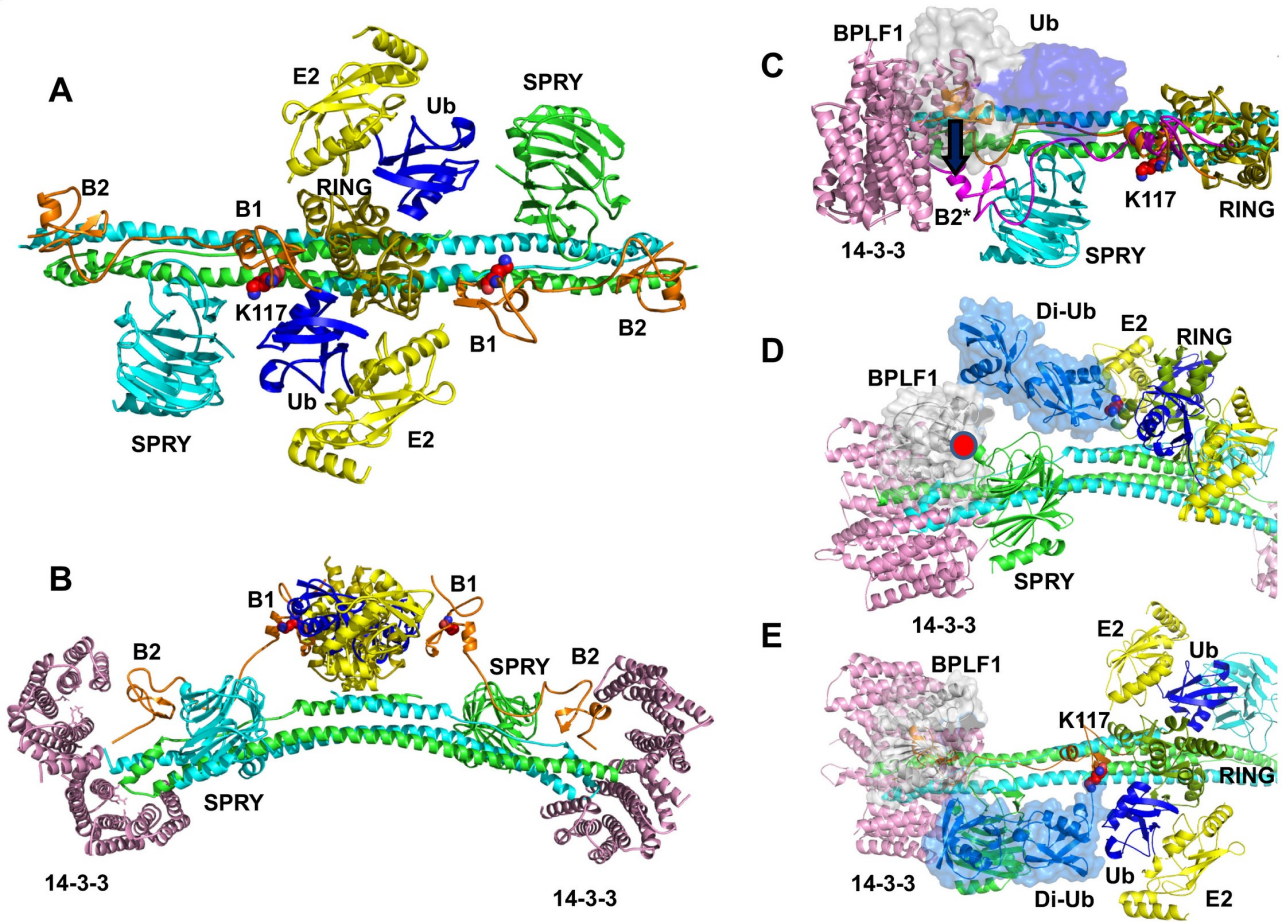
Predicted mode of regulation of TRIM25 by 14-3-3 and BPLF1. **A.** Top view of the TRIM25 dimer (green and cyan) in complex with the ubiquitin (blue) loaded E2 (yellow). The long linker with interposed B-box1 and B-box2 connecting the juxtaposed RING domains (olive) to the opposite ends of the CC dimer is colored orange. The autoubiquitination site on B-Box1 K117 is highlighted. **B.** Side view of TRIM25:E2 complex bound to two 14-3-3 dimers (pink). Interaction of the tip of the CC domain with one binding pockets of the 14-3-3 dimer would leave the second pocket available for interaction with additional partners. **C**. Side view of the TRIM25:14-3-3:BPLF1 complex. BPLF1 (grey) and the bound ubiquitin (blue) are space filled. The interaction of BPLF1 with the binding groove of 14-3-3 orients the catalytic site of BPLF1 towards the TRIM25 autoubiquitination site on B-box1 (filled spheres) and causes a large displacement of the B-Boxes. **D.** Side view of the di-ubiquitinated complex illustrating the distance between the K48-linked di-ubiquitin and catalytic site of BPLF1. **E.** Top view of the di-ubiquitinated complex illustrating the potential interference of the di-ubiquitin chain with the position of the SPRY:substrate complex.

Since a crystal structure of the full-length TRIM25 is not available, a molecular model of the entire molecule was generated from the crystal structures of the individual domains [21, 30, 43] (Fig 10). The model offers interesting clues on the regulation of TRIM25 (Fig 10 and S5 Fig, animation). The long and disordered loops that separate B-box1 from the RING domain (80–105 residues), the two B-boxes (25 residues), and the PRY-SPRY from the CC domain (65 residues) allow ample mobility that could be of a great importance for the activity of the ligase. A reconstruction of the TRIM25 dimer bound to the ubiquitin loaded E2 is shown in Fig 10A (top view) and 10B (side view). The flexible connections between the juxtaposed RINGs and the opposite ends of the CC dimers may allow rotation of the RING:E2 complex, positioning the E2 in correct orientation for the discharge of ubiquitin either onto the autoubiquitination site in B-box1-K117 (red spheres), or the substrate bound to the PRY-SPRY domain. Docking of the tip of the CC domain to one binding site in the pocket of the 14-3-3 dimer would leave the second site available for interaction with additional partners. Upon binding of two TRIM25 dimers, this may promote the formation of large supramolecular complexes that are required for optimal catalysis. The recruitment of BPLF1 to the 14-3-3:TRIM25 complex may activate the ligase by promoting a displacement of the B-boxes and PRY-SPRY domains that mimic the recruitment of a second TRIM25 dimer or the binding of substrate on the PRY-SPRY domain (Fig 10C). Binding to 14-3-3 orients the catalytic site of BPLF1 towards the autoubiquitination site but the distance between the catalytic pocket and B-box1 may not allow removal of the first, or first and second conjugated ubiquitins (Fig 10D). Mono/di-ubiquitination will allow more mobility of the B-box1 compared to polyubiquitination, which may hinder the correct positioning of substrates bound to the PRY-SPRY domain towards the E2, contributing thereby to inactivation of the ligase (Fig 10E).

## Discussion

The TRIM family of ubiquitin ligases plays a pivotal role in the activation of antiviral responses [44] and various members of the family are targeted by viruses to enable evasion from the host immune control [15]. The importance of TRIMs in the regulation of innate antiviral immunity is clearly illustrated by the capacity of TRIM25 to promote the IFN response via ubiquitination of the RNA sensor RIG-I [16]. Our study reveals a novel mechanism by which the viral deconjugases encoded by herpesviruses co-opt the 14-3-3 molecular scaffolds to regulate the activity of TRIM25, and provides a first insight on the molecular interactions involved in the physiological activation and viral-induced inactivation of the ligase.

BPLF1 is the EBV-encoded member of the large tegument proteins of herpesviruses whose deconjugase activity plays important roles in the biology of virus infection and may also contribute to oncogenesis [45]. Previous studies have shown that the inhibition of RIG-I ubiquitination induced by catalytically active BPLF1 correlates with the formation of a trimolecular complex including TRIM25 and the 14-3-3 molecular scaffold [26] but the relationship between these effects was not understood. Here we have found that expression of catalytically inactive BPLF1 activates TRIM25 and promotes K48-linked autoubiquitination and degradation of the ligase (Figs 1, 2 and S2). It was previously shown that TRIM25 forms stable dimers *in vitro* via pairing of two CC domains in antiparallel orientation while activation of the ligase requires juxtaposition of the RING domains, which is in turn dependent on stabilizing interactions provided by the conjugating enzyme, and possibly by the substrate [9, 30]. Our findings suggest that the recruitment of BPLF1 could activate the ligase by promoting a conformational rearrangement that mimics substrate binding. Interestingly, this unconventional activation is associated with the attachment of K48-linked ubiquitin chains and enhanced TRIM25 degradation but does not appear to prevent the activity of the ligase towards conventional substrates since the IFN response is not inhibited and RIG-I is efficiently ubiquitinated in cells expressing inactive BPLF1 [26] (Fig 5E). This is in line with previous reports that have identified a dominant TRIM25 autoubiquitination site on residue K117 in B-box1, and demonstrated that, in spite of accelerated turnover, autoubiquitination does not interfere with the function of the ligase [18].

In contrast to the polyubiquitination induced by mutant BPLF1, expression of the catalytically active viral DUB was associated with trimming of the TRIM25 polyubiquitin chains to mono- or di-ubiquitin (Fig 1). Mono/di-ubiquitinated TRIM25 showed a slower turnover and was sequestered into aggregates where it co-localized with the autophagy receptor p62/SQSTM1 (Figs 1E and 2D). It is noteworthy that the expression of BPLF1 was sufficient for the formation of TRIM25 aggregates, independently of IFN triggering. Furthermore, the aggregates differed from the stress granules induced by viral infection in their failure to colocalize with SG markers such as TIA1 (Fig 2B) and to recruit endogenous RIG-I (Fig 2C). The colocalization with p62/SQSTM1 is consistent with the accumulation of mono/di-ubiquitinated TRIM25 and suggest that, while becoming resistant to proteasome degradation due to trimming of the polyubiquitin chains, the ligase may be destined for degradation by autophagy. However, degradation appears to be halted since catalytically active BPLF1 promoted the stabilization of TRIM25 (Fig 1E). This, together with our previous finding that the BPLF1 interactome includes several members of the autophagy machinery [26], suggests that catalytically active BPLF1 may inhibit autophagy; a possibility currently under investigation. Interestingly, TRIM25 aggregates were also detected in the majority of EBV positive cells upon induction of the productive virus cycle (Fig 4). These aggregates resemble those observed in BPLF1 transfected cells in their failure to co-localize with TIA1 but, in line with our previous finding that BPLF1 does not affect the binding of TRIM25 to activated RIG-I [26], the majority of the aggregates were stained with a RIG-I specific antibody.

Given the correlation between the formation of TRIM25 aggregates and inhibition of the IFN response it was important to dissect the molecular interaction involved in this effect. We found that a catalytically active BPLF1 helix-2 mutant that fails to bind to 14-3-3 was also unable to induce the formation of aggregates and inhibit the IFN response (Figs 4, 5 and 6), pointing to the interaction with 14-3-3 as key mediator of signal inactivation. 14-3-3 molecular chaperones regulate the activity of several ubiquitin ligases with both enhancing and inhibitory effects [46–49]. Members of the 14-3-3 family bind to TRIM25 either constitutively or selectively upon induction of the IFN response [27], but the sites and modes of interaction have not been explored. It is noteworthy that the BPLF1 helix-2 mutant that failed to interact with 14-3-3 retained both catalytic activity (S3 Fig) and the capacity to bind to TRIM25 suggesting that, without the stabilizing effect of 14-3-3, the interaction between the viral DUB and the ligase is either too weak or unable to position the catalytic site of BPLF1 in a functionally relevant orientation relative to the substrate. *In vitro* binding assays using isolated TRIM25 domains and bacterially expressed 14-3-3 and BPLF1 have provided a first insight on the molecular interactions that may be involved in both the activation and inactivation of the ligase. We found that 14-3-3 interacts with the CC domain of TRIM25 while BPLF1 contacts both the CC and B-box domains, pointing to the opposite ends of the CC dimer as possible sites of assembly of the trimolecular complex (Figs 7, 8, 9 and 10). All 14-3-3 family members form homo- or heterodimers enclosing a conserved binding groove with two symmetrically oriented binding pockets [50]. Structural comparison of all family members bound to their specific ligands suggests that conformational flexibility in both the substrate binding groove and the dimer interface may allow adaptation to different types of ligands, whether phosphorylated and extended or non-phosphorylated and helical, which could facilitate the interaction with proteins of varying size and sequence [41]. Our docking model suggests that acidic residues in BPLF1 helix-2 and TRIM25-CCD could interact with a cluster of highly conserved basic residues that are present in the crevice of all 14-3-3 isoforms (Fig 9). The flexibility of the groove could allow simultaneous interaction with both the slim tip of the CC dimer and the bulky helix-2 of BPLF1, positioning the catalytic site of the DUB close to the putative autoubiquitination site in B-box1 (Fig 10). Interestingly, the molecular model predicts that the presence of BPLF1 would not interfere with the capacity of the PRY-SPRY domain to position the substrate in correct orientation towards the conjugating enzyme bound to the RING domain (Fig 10). Thus, the long flexible linkers connecting the RING:E2 dimer to the opposite ends of the CC dimer may allow both autoubiquitination and ubiquitination of the substrate, as observed in cells expressing catalytically inactive BPLF1. The attachment to B-box1 of long polyubiquitin chains pointing away from the complex may not interfere with ubiquitination of the substrate, whereas the presence of only one or two conjugated ubiquitins will allow higher mobility, which may hinder correct positioning of the substrate bound to the PRY-PSRY domain and contribute to inactivation of the ligase.

Our finding that the interaction with 14-3-3 is critical for the capacity of BPLF1 to inhibit the IFN response offers an interesting insight on the molecular features that underlie the function of TRIM25 and the link between self-association and catalytic activity. While TRIM25 dimers are catalytically active, oligomerization appears to be required for efficient substrate ubiquitination [30, 43]. By bringing together two TRIM25 dimers, 14-3-3 could induce the formation of supramolecular complexes, providing a molecular explanation for the capacity of 14-3-3 to facilitate the ubiquitination and MAVS translocation of activated RIG-I [27]. It is noteworthy that while the 14-3-3ε isoform was shown to form inducible complexes with TRIM25 in Sendai virus infected hepatoma cells, 14-3-3η and 14-3-3σ exhibited constitutive TRIM25 binding [27]. We did not specifically address the 14-3-3 isoform involved in this induction of IFN response in EBV infected cells but show that BPLF1 interacts with the conserved binding pocket of 14-3-3 which implies that all isoforms are potential partners. Interestingly, BPLF1 appears to bind more efficiently to 14-3-3η isoform and may in so doing stabilize a 14-3-3η:TRIM25 complex that is not proficient in IFN signaling. Further studies will be required to explore the intriguing possibility that different 14-3-3 isoforms may have different regulatory effects on the IFN response depending on the type of viral infection or virus infected cell.

In conclusion, our study provides a compelling example of how viruses may inhibit the IFN response by co-opting a family of cellular proteins that regulate the activity of the TRIM ubiquitin ligases. While attempts to validate the predicted interactions by X-ray crystallography and cryo-EM are currently in progress, the model presented in our study offers a first molecular insight on the mechanism of activation of TRIM25 by 14-3-3 and inactivation of the ligase by the viral DUB. Since both 14-3-3 and BPLF1 interact with several cellular proteins [26], these observations should stimulate the search for additional regulatory complexes that may play specific roles in the cellular remodeling that enables the establishment of latent EBV infections and virus reactivation.

## Materials and methods

### Chemicals

DL-Dithiothreitol (DTT, D0632), N-Ethylmaleimide (NEM, E1271), Iodoacetamide (I1149), IGEPAL CA-630 (NP40, I3021), Triton X-100 (T9284), bovine serum albumin (BSA, A7906), Sodium dodecyl sulfate (SDS, L3771), Tween-20 (P9416), Cycloheximide solution (C4859), Glutathione (G4251), Ethylenediaminetetraacetic acid disodium salt dehydrate (EDTA, E4884) and Trizma base (Tris, 93349) Sodium butyrate (NaBu, B5887) and 12-O-tetradecanoykphorbol-13-acetate (TPA, 4174) were purchased from Sigma-Aldrich (St. Louis, MO, USA). Complete protease inhibitor cocktail (04693116001), and phosphatase inhibitor cocktail (04906837001) were purchased from Roche Diagnostic (Mannheim, Germany). Ciprofloxacin (17850) was purchased from Fluka (Buchs, Switzerland). Poly(I:C) (LMW)/LyoVec was purchased from Invivogen (San Diego, CA, USA).

### Antibodies

Antibodies and their manufacturers were: mouse anti-β-actin clone AC-15 (1:5000, A5441), mouse anti-FLAG clone M2 (1:5000, IF: 1:500; F1804) from Sigma-Aldrich; goat anti-FLAG (IF 1:500; ab1257) and rabbit anti TRIM25 clone EPR7315 (1:2000; ab167154) from Abcam (Cambridge, MA, USA); mouse anti-HA clone 12CA5 (1:2000; 11583816001) from Roche (Mannheim, Germany); rabbit anti-ubiquitin (1:1000; Z0458) from Dako (Glostrup, Denmark); rabbit K48-specific anti-ubiquitin clone Apu2 (1:1000; 05–1307) mouse anti-RIG-I clone 1C3 (WB 1:1000; IF 1:100, MABF297) from EMD-Millipore (Darmstadt, Germany); goat anti-TIA-1 clone C20 (IF 1:200); mouse anti-C-Myc clone 9E10 (1:1000; sc-40), mouse anti-pan14-3-3 clone H-8 (1:1000; sc-1657), rabbit anti-14-3-3β clone C20 (1:1000; sc-628), rabbit anti-14-3-3γ clone C-16 (1:1000; sc-731), rabbit anti-14-3-3θ clone C-17 (1:1000; sc-732), rabbit anti-14-3-3ζ clone C-16 (1:1000; sc-1019) and rabbit anti-14-3-3ε clone T-16 (1:1000; sc-1020) from Santa-Cruz Biotechnology (Santa Cruz, CA, USA); rabbit anti-14-3-3η clone D23B7 (1:1000; #5521), rabbit anti-MDA5 clone D74E4 (1:1000; #5321), rabbit anti-RIG-I clone D14G6 (1:1000; #3743), rabbit anti-IRF-3 clone D6I4C (1:1000, IF 1:400; #11904), rabbit anti-pIRF-3 clone 4D4G (1:1000; #4947) and mouse anti-GST clone 26H1 (1:1000, IF: 1:800; #2624) from Cell-Signaling Technologies (Danvers, MA, USA); mouse anti-V5 clone 2F11F7 (1:2000; 37–7500) from Invitrogen (Rockford, IL, USA); mouse anti-HA.11 clone 16B12 (1:1000; 901501) from BioLegend (San Diego, CA, USA). Mouse anti p62/SQSTM1 (IF 1:200, 610823) was from Becton Dickinson (San Jose, CA, USA). Alexa Fluor rabbit-488, mouse-555, rabbit-555 and goat-647 conjugated secondary antibodies raised in donkey (A21206, A31570, A31572 and A21447, respectively); rabbit-568 and mouse-647 conjugated secondary antibodies raised in goat (A11011 and A21236, respectively) were from Thermo Fisher (Waltham, MA, USA).

### Plasmids

Plasmids encoding 3xFLAG-BPLF1 (amino acid residues 1–235), the BPLF1-C61A, the BPLF1-D86-90R mutants and KSHV-ORF64 were described previously [35, 51]. Plasmids encoding for GST-2CARD, V5-TRIM25 and V5-TRIM25ΔRING were kindly provided by Jae Jung, University of Southern California, USA. The pcDNA3.0-HA-TRIM25 plasmid (Addgene plasmid # 12452) was a gifts from Dong-Er Zhang [52]. Plasmids encoding GST-TRIM25-RING, GST-TRIM25-RING-BB, GST-TRIM25-BB, GST-TRIM25-CC and GST-TRIM25-SPRY [23] were kindly gifted by Michaela Gack, University of Chicago, USA. Plasmids encoding Myc-14-3-3η and the 14-3-3η K50E and V181D mutants were kindly gifted by Zhenyu Yue, Mount Sinai School of Medicine, NY, USA [37]. A synthetic His-tagged 14-3-3η coding sequence (Geneuniversal, DE, USA) was cloned between the BamHI and XhoI restriction sites of the pET28a(+) vector. A DNA fragment coding amino acid 1–274 of BPLF1 was amplified from the 3xFLAG-BPLF1 vector using the primers 5´-TCACAGCAGCGGCC TGATGAGTAACGGCGACTG-3’ and 5-’CGAGTGCGGCCGCAAGTTACACAAGCTCG GGCC-3’ and the pET28 prokaryotic-expression plasmid was amplified using the primers 5’-CAGGCCGCTGCTGT-3’ and 5’-CTTGCGGCCGCAC-3’. Cloning was performed following the FastCloning technique protocol [53] and verified by sequencing.

### Cell lines, transfection and EBV reactivation

HeLa cells (ATCC RR-B51S) were cultured in Dulbecco’s minimal essential medium (DMEM, Sigma-Aldrich), supplemented with 10% FCS (Gibco-Invitrogen), ciprofloxacin (10 μg/ml) and maintained in a 37°C incubator in 5% CO_2_. EBV converted AGS-Bx1 cell line (kindly provided by Alan Chiang, Hong Kong University, Hong Kong) [54] were cultured in Dulbecco's Modified Eagle Medium: Nutrient Mixture F-12 (DMEM/F12) (GIBCO-Invitrogen, Carlsbad, USA) supplemented with 10% Fetal Bovine Serum and 500 μg/ml Geneticin (GIBCO-Invitrogen). Plasmid transfection was performed using the JetPEI DNA transfection reagent (Polyplus transfection; Illkirch, France) as recommended by the manufacturer. Low molecular weight (LMW) Poly (I:C) was transfected using the transfection reagent LyoVec as Poly(I:C) (LMW) / LyoVec (InvivoGen; Toulouse, France) as per manufacturers guidelines. To induce the productive virus cycle AGS-Bx1 cells were cultured for 24 h in medium supplemented with 30 ng/ml TPA and 0.5 mM NaBu.

### Immunoblotting and immunoprecipitation

Cells harvested 48 h post transfection were lysed in NP-40 lysis buffer (50 mM Tris-HCl pH 7.6, 150 mM NaCl, 5 mM MgCl2, 1 mM EDTA, 1 mM DTT, 1% Igepal, 10% glycerol) supplemented with protease inhibitor cocktail 20 mM NEM and 20 mM Iodoacetamide and phosphatase inhibitor cocktail whenever required. Protein concentration was measured with a protein assay kit (Bio-Rad Laboratories). For BPLF1 co-immunoprecipitation, the cell lysates were incubated for 4 h with anti-FLAG agarose affinity gel (A-2220; Sigma), followed by washing with lysis buffer and elution with 300 μg/ml of the FLAG peptide (F4799; Sigma). For co-immunoprecipitation of endogenous TRIM25 the cell lysates were incubated for 4 h with a rabbit anti-human TRIM25 specific antibody followed by 2 h with protein-G coupled Sepharose beads (GE Healthcare). The beads were washed with lysis buffer and elution was performed by boiling for 10 min in 2x SDS-PAGE loading buffer. To asses ubiquitination, ectopically expressed TRIM25 was immunoprecipitated using either anti-HA-agarose (Clone HA-7, A2095; Sigma) anti-V5-agarose (Clone V5-10, A7345; Sigma) or mouse anti-human TRIM25 and endogenous RIG-I was immunoprecipitated with rabbit anti-human RIG-I specific antibody under denaturing conditions. Cell pellets were lysed in 100 μl NP-40 lysis buffer (50 mM Tris-HCl pH 7.6, 150 mM NaCl, 1 mM EDTA, 1mM DTT, 1% Igepal) supplemented with 1% sodium dodecyl sulfate (SDS). Before immunoprecipitation NP-40 buffer was added to reach a final concentration of 0.1% SDS. The beads were washed with lysis buffer containing 0.1% SDS. Elution was performed by either using HA-peptide (I2149, Sigma) or by boiling with 2x SDS-PAGE loading buffer. Equal amounts of proteins were fractionated in polyacrylamide Bis-Tris 4–12% gradient gels (Invitrogen). After transfer to poly-vinylidene difluoride (PVDF) membranes (Millipore), the blots were blocked in Tris-buffered saline containing 5% non-fat skimmed milk powder and 0.1% Tween-20 and incubated with primary antibodies for either 1 h at room temperature or overnight at 4°C, followed by incubation for 1 h with the appropriate horseradish peroxidase-conjugated secondary antibodies. The complexes were visualized by chemiluminescence (ECL; GE Healthcare).

### Protein expression and purification

Poly-histidine-tagged proteins were expressed in *E*.*coli* BL21-DE3 induced with 1 mM IPTG for 3 h and purified using Ni-NTA resin (QIAGEN) according to the manufacturer’s instruction. 6xHis-BPLF1 was further subjected to gel filtration using a Superdex 75 10/300 GL column (GE Healthcare Life Sciences). The purified protein was eluted as a monomer in 20 mM Tris-HCl pH 7.5 and 150 mM NaCl. GST-tagged TRIM25 domains were expressed in transfected HeLa cells. After wash in cold PBS, the cells were lysed in buffer containing 50 mM Tris-HCl, pH 7.6, 150 mM NaCl, 1% Igepal, 0.1% SDS, 1mM EDTA, 1 mM DTT and protease inhibitor cocktail. GST-tagged proteins were purified with glutathione-sepharose 4B beads (GE Healthcare) and eluted with 40 mM glutathione.

### Protein binding assay

Purified His- and GST-tagged proteins were incubated for 20 mins at 4°C in binding buffer (50 mM Tris–HCl, 100 mM NaCl, 1 mM DTT, 0.5% Igepal) followed by capture of the complexes by incubation with 25 μL packed volume of prewashed Ni-NTA beads (Qiagen) for 20mins at 4°C with gentle rotation. After extensive washing, the proteins were eluted using 300 mM imidazole in buffer containing 50 mM Tris-HCl pH 7.6, 50 mM NaCl and 1 mM DTT.

### Assay of protein turnover

To determine the effect of BPLF1 on the turnover of endogenous TRIM25, HeLa cells were transfected with either empty vector or Flag-BPLF1 plasmids. Twenty-four hours post transfection the cells were treated with 100 μg/ml CHX and aliquots were harvested at the indicated times. Equivalent amounts of proteins were resolved by SDS-PAGE and analyzed by Western blotting.

### Immunofluorescence and confocal microscopy

HeLa cells were grown to semi-confluency in Dulbecco’s minimal essential medium containing 10% fetal calf serum and 100 μg/ml ciprofloxacin on glass cover slips and transfected with the indicated plasmids using the JetPEI kit as recommended by the manufacturer. AGS-Bx1 cells were grown to near confluency in Dulbecco's Modified Eagle Medium: Nutrient Mixture F-12 (DMEM/F12) (GIBCO-Invitrogen, Carlsbad, USA) supplemented with 10% Fetal Bovine Serum on glass coverslip and induced for productive cycle with TPA/NaBu. After 24 h the cells were fixed in 4% paraformaldehyde (Merck, 100496). To stain endogenous TRIM25, RIG-I and TIA-1 the cells were permeabilized with 0.05% Triton X-100 in PBS for 5 min at room temperature (RT), blocked with 3% BSA in PBST (0.05% Triton X-100 in PBS) for 30 min, and labelled with rabbit anti-TRIM25, rabbit anti-RIG-I, goat anti-TIA-1 or mouse anti-FLAG antibodies diluted in blocking buffer. To stain p62/SQSTM1 and endogenous IRF3 in GST-2CARD activated cells, the cells were permeabilized using 0.1% Triton X-100 in PBS, followed by blocking with 0.12% glycine (Fisher Scientific, G46-1) in PBS for 10 min, and 3% bovine serum albumin (BSA, Sigma, A7906) in PBS for 15 min at room temperature. The cells were triple labeled in 3% BSA-PBS using rabbit anti-IRF3, mouse anti-GST and goat anti-FLAG, or rabbit anti-TRIM25, mouse anti-p62 and goat anti-FLAG antibodies and then incubated with appropriate Alexa Fluor 488, 555 or 647 conjugated secondary antibodies. The coverslips were mounted cell side down on object glasses with Mowiol (Calbiochem, 475904) containing 50 mg/ml 1,4-diazabicyclo[2.2.2]octane (Dabco; Sigma, D-2522) as anti-fading agent and 2 μg/ml Hoechst 33258 (Sigma, 861405), or Vectashield mounting medium with DAPI (Vector laboratories, H-1200) to stain the nuclei. The samples were imaged using a confocal scanning laser microscope (Zeiss LSM800 META) and 1 or 2.5 μm optical sections were acquired.

### Quantitative RT-PCR

Messenger RNA expression of components of the RIG-I signaling pathway and human GAPDH was measured by qRT-PCR using the primers: IFNβ 5’-TCCAA ATTGCTCTCCTGTTG-3’ (sense), 5’-GCAGTATTCAAGCCTCCCAT-3’ (antisense); RIG-I 5’-ATCCCAGTGTATGAACAGCAG-3’ (sense), 5’-GCCTGTAACTCTATACCCATGTC-3’ (antisense); MDA5 5’-TGGTCTCGTCACCAATGAAA-3’ (sense), 5’-CTCCTGAACCAC TGTGAGCA-3’ (antisense); GAPDH 5’-TGGGCTACACTGAGCACCAG-3’ (sense), 5’-GG GTGTCGCTGTTGAAGTCA-3’(antisense). Total RNA was isolated using the RNeasy Mini Kit (Qiagen, Hilden, Germany) and reverse transcribed using a high capacity reverse transcription kit (Applied Biosystems, CA, USA) for 10 min at 25°C, followed by 37°C for 120 min and 85°C for 5 min. Quantitative RT-PCR assays were setup using the Power SYBR Green PCR Master Mix (Applied Biosystems by Life Technologies, Woolston Warrington, UK) using 100 nM of primer pairs with cycling conditions: initial 50°C 2 min, denaturation 95°C 10 min, followed by 40 cycles of 95°C for 15 sec, 60°C for 1 min. Melting curves were run by incubating the reaction mixtures at 95°C for 15 sec, 60°C for 20 sec, 95°C for 15 sec, ramping from 60°C to 95°C in 1°C/sec. The values were normalized to endogenous GAPDH. Fold change was calculated as: Fold Change = 2-Δ(ΔCt) where ΔCt = Ct target—Ct housekeeping and Δ(ΔCT) = ΔCt vector treated - ΔCt BPLF1 treated, according to the Minimum Information for Publication of Quantitative Real-Time PCR Experiments (MIQE) guidelines.

### Molecular modeling

A structural model of the DUB module of BPLF1 was created based on the crystal structure of the DUB module of murine cytomegalovirus (MCMV) M48 (PBD code 2J7Q, [42]) using the homology modeling option of SwissModel (https://www.swissmodel.expasy.org/) [55]. Docking of TRIM25, 14-3-3 and BPLF1 was performed using the ClusPro docking server (https://cluspro.bu.edu/login.php) [56]. The crystal structure of the CCD-SPRY domain of TRIM25 (PDB code 6FLN) [21] was used as a receptor and the crystal structure of 14-3-3 in open conformation (PDB code 2C23) [41] was used as a ligand. Docking was done using restrained distance between V181 in 14-3-3 and E303 in the TRIM25 CC domain ranging between 2-5Å [57]. Two highly populated clusters of TRIM25/14-3-3 binary complexes with more than 85 members were used for docking the homology model of BPLF1 bound to ubiquitin to exclude docking results with the active site buried in the interface of the complex. A distance between 14-3-3-V181 and BPLF1-D90 ranging between 3-7Å was used as restraint. The third top model of the docking output that predicted the active site of BPLF1 turned towards the TRIM25:14-3-3 complex was used for further analysis. A theoretical model of the full-length TRIM25 was created by combining the crystal structures of the RING/E2/Ub complex (PDB code 5FER) [30], the modified tandem B-boxes in extended conformation (PDB code 2JUN) [58] and the coiled-coil/SPRY domains (PDB code 6FLN) [59]. The crystal structure of human 14-3-3η (PDB code 5YQG) [60] was used to model the TRIM25:14-3-3 complex. The crystal structure of K48-linked di-ubiquitin (PDB code 5GOI) [61] was used to model auto-ubiquitination of TRIM25 at K117 of B-Box1. Modeling and preparation of the structural figures were performed using the PyMol software (PyMol Molecular Graphics System, Version 2.3.0 Schrödinger, LLC).

## Supporting information

S1 Fig Expression of catalytically inactive BPLF1 induces K48-linked auto-ubiquitination of endogenous TRIM25. The indicated FLAG-tagged plasmids were co-transfected in HeLa cells and endogenous TRIM25 was immunoprecipitated under denaturing conditions. The immunoprecipitates were probed with the ubiquitin-specific antibody. One representative experiment out of two is shown in the figure.
(TIF)

S2 Fig Catalytically active and inactive BPLF1 have different effects on the subcellular localization of TRIM25. **A.** Representative micrographs illustrating the failure of TRIM25 to form aggregates in cells expressing catalytically inactive BPLF1. **B.** Quantification of the number of BPLF1/BPLF1-C61A positive cells exhibiting TRIM25 aggregates. A small number of stress granules identified by colocalization with TIA-1 (see Fig 2B) were observed in transfected cells independently of BPLF1 expression. The mean ± SD of three independent experiments is shown. Statistical analysis was performed using Student's *t*‐test, ***P ≤ 0.001. **B.** Quantification of TRIM25 specific fluorescence in cells expressing catalytically inactive BPLF1-C61A. HeLa cells transfected with FLAG-BPLF1-C61A were stained 24 h after transfection with FLAG and TRIM25 specific antibodies. Fluorescence intensity was quantified using the ImageJ software in transfected cells that did or did not express BPLF1-C61A. The mean ± SD of three independent experiments where ≥100 cells were scored is shown. Statistical analysis was performed using the Student's *t*‐test, ***P ≤ 0.001.
(TIF)

S3 Fig The BPLF1-D86-90R mutant binds to TRIM25 and is catalytically active but fails to induce TRIM25 autoubiquitination. **A.** The BPLF1-D86-90R mutant is catalytically active. The enzymatic activity of FLAG-tagged BPLF1, the D86-90R binding mutant and C61A catalytic mutant was assessed by labeling with Ub-VS and Ub-VME functional probes and visualized in western blot probed with the anti-Flag antibodies. Crosslinking of the probe to the active enzymes caused a band shift of approximately 10 kD. **B**. An excess of the BPLF1-D86-90R binding mutant is still unable to promote TRIM25 auto-ubiquitination. HA-TRIM25 was co-transfected with either BPLF1-D86-90R or different amounts of catalytically active BPLF1 and western blots were probed with the HA antibody. A band shift corresponding to monoubiquitinated TRIM25 was detected in the cells even upon transfection of a small amount of wild type BPLF1 but not in cells transfected with a 12-fold higher amount of the BPLF1-D86-90R mutant.
(TIF)

S4 Fig The binding of 14-3-3 to TRIM25 and BPLF1 is not dependent on phosphorylation. **A.** Cell lysates were prepared from Hela cells transfected with FLAG-BPLF1 in the presence or absence of phosphatase inhibitors. FLAG and TRIM25 immunoprecipitates were probed with antibodies to 14-3-3. One representative western blot out of three is shown in the figure. **B**. The intensity of the 14-3-3 specific bands were quantified by densitometry. Relative binding is expressed as the ratio of the intensity of the 14-3-3 band in the presence or absence of phosphatase inhibitors. The mean ± SD of three experiments is shown in the figure.
(TIF)

S5 Fig Animation of the reconstructed 14-3-3:TRIM25:BPLF1 trimolecular complex. Color coding as in Fig 10.
(MP4)
