# Supplementary figures and images for: 14-3-3 scaffold proteins mediate the inactivation of trim25 and inhibition of the type I interferon response by herpesvirus deconjugases
Source: PLoS Pathog. 2019 Nov 11;15(11):e1008146. doi: 10.1371/journal.ppat.1008146 (PMC6874091; doi:10.1371/journal.ppat.1008146)

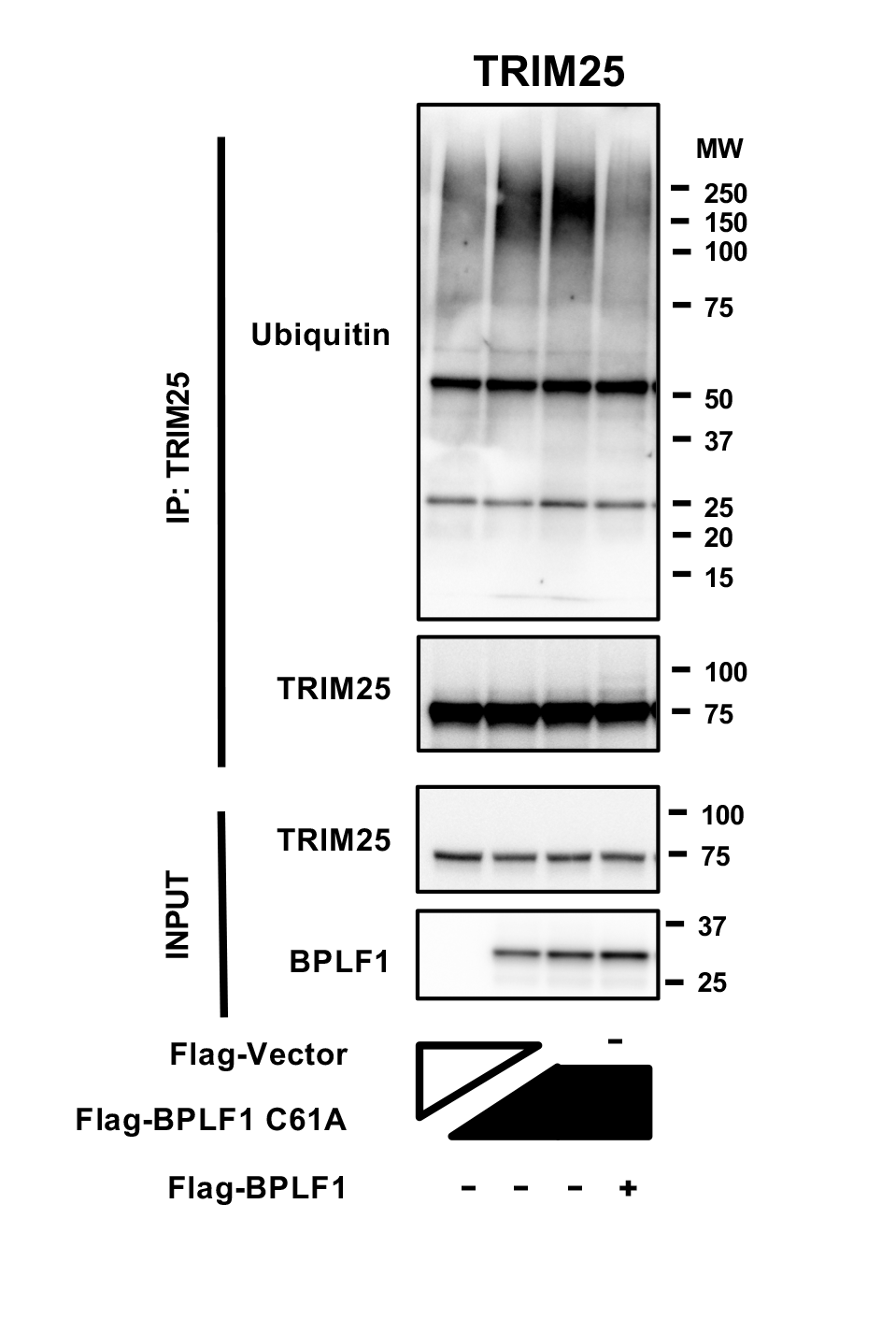

Supplement: S1 Fig — The indicated FLAG-tagged plasmids were co-transfected in HeLa cells and endogenous TRIM25 was immunoprecipitated under denaturing conditions. The immunoprecipitates were probed with the ubiquitin-specific antibody. One representative experiment out of two is shown in the figure. (TIF) [file ppat.1008146.s001.tif]

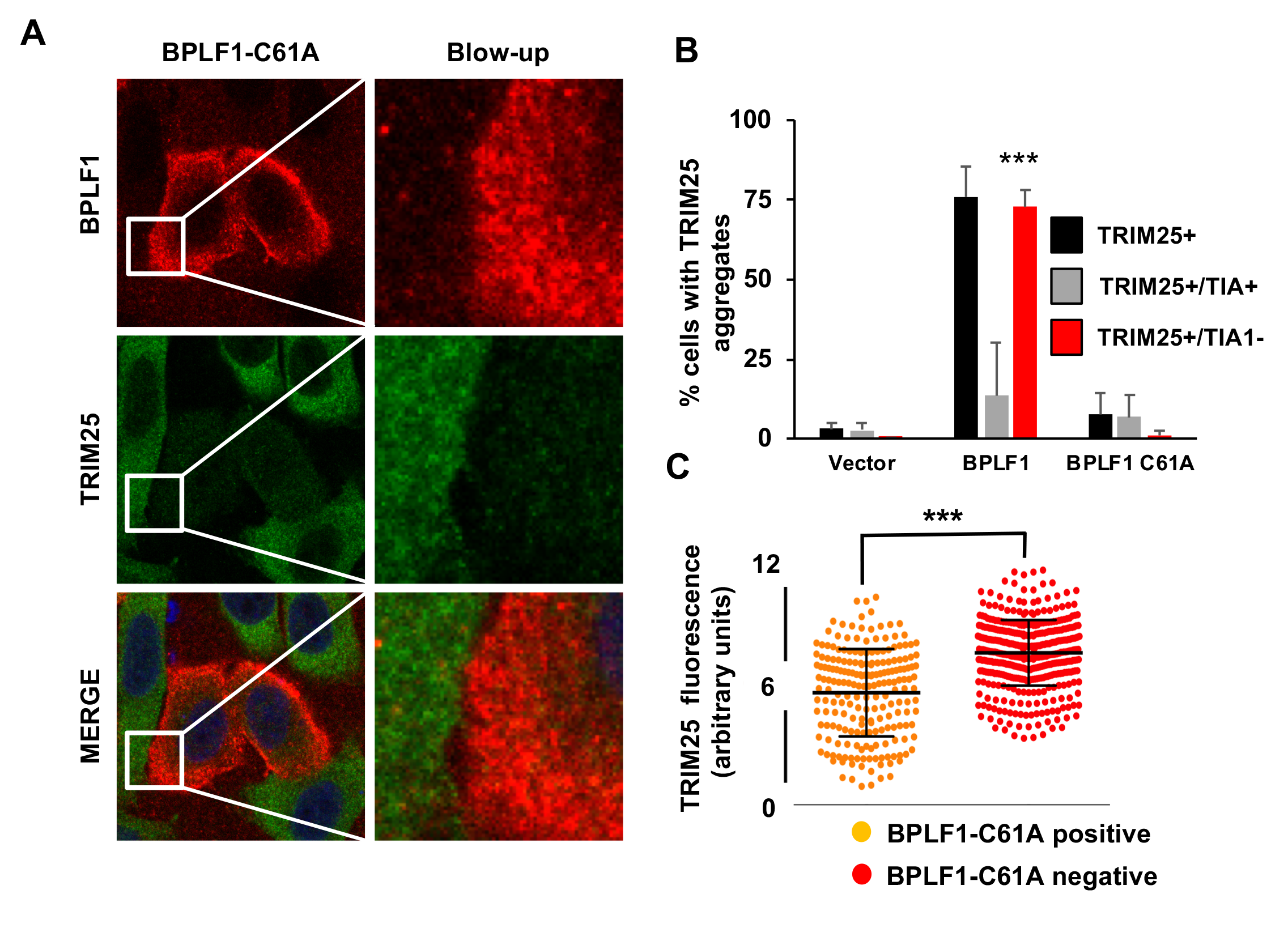

Supplement: S2 Fig — A. Representative micrographs illustrating the failure of TRIM25 to form aggregates in cells expressing catalytically inactive BPLF1. B. Quantification of the number of BPLF1/BPLF1-C61A positive cells exhibiting TRIM25 aggregates. A small number of stress granules identified by colocalization with TIA-1 (see Fig 2B) were observed in transfected cells independently of BPLF1 expression. The mean ± SD of three independent experiments is shown. Statistical analysis was performed using Student's t‐test, ***P ≤ 0.001. B. Quantification of TRIM25 specific fluorescence in cells expressing catalytically inactive BPLF1-C61A. HeLa cells transfected with FLAG-BPLF1-C61A were stained 24 h after transfection with FLAG and TRIM25 specific antibodies. Fluorescence intensity was quantified using the ImageJ software in transfected cells that did or did not express BPLF1-C61A. The mean ± SD of three independent experiments where ≥100 cells were scored is shown. Statistical analysis was performed using the Student's t‐test, ***P ≤ 0.001. (TIF) [file ppat.1008146.s002.tif]

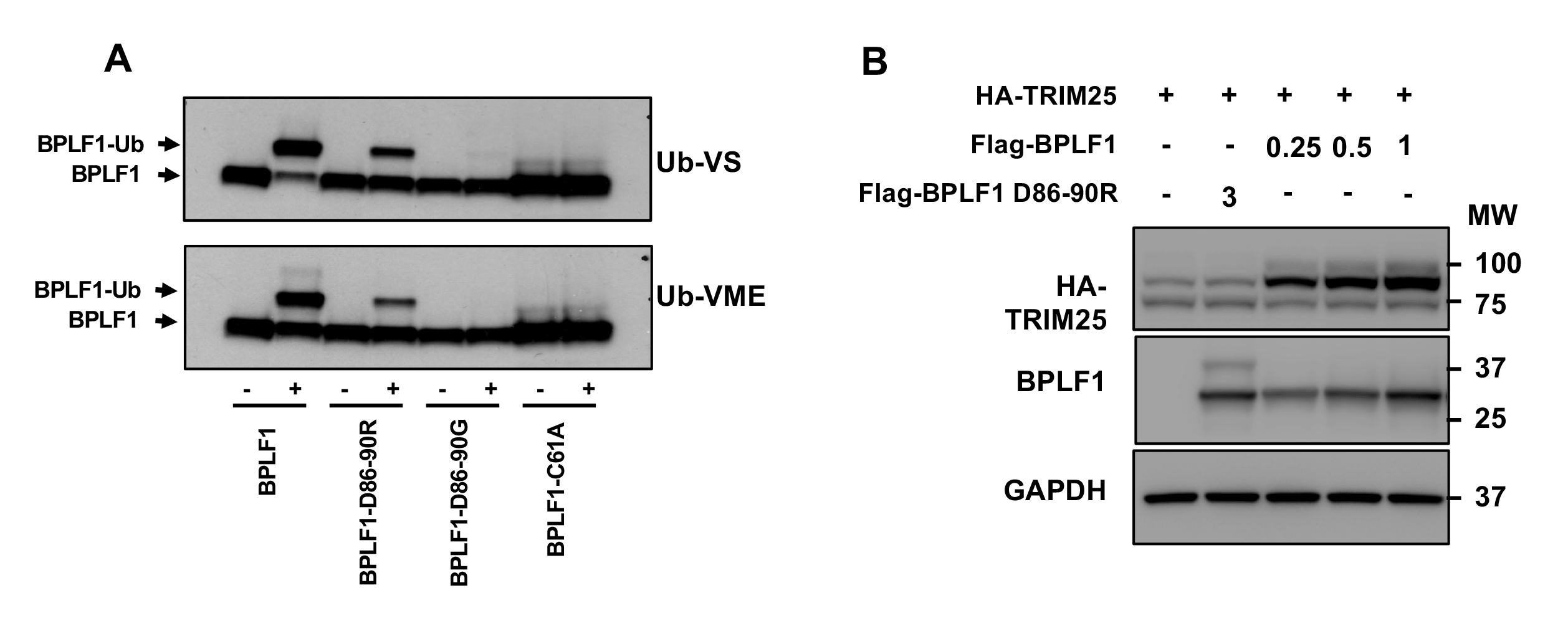

Supplement: S3 Fig — A. The BPLF1-D86-90R mutant is catalytically active. The enzymatic activity of FLAG-tagged BPLF1, the D86-90R binding mutant and C61A catalytic mutant was assessed by labeling with Ub-VS and Ub-VME functional probes and visualized in western blot probed with the anti-Flag antibodies. Crosslinking of the probe to the active enzymes caused a band shift of approximately 10 kD. B. An excess of the BPLF1-D86-90R binding mutant is still unable to promote TRIM25 auto-ubiquitination. HA-TRIM25 was co-transfected with either BPLF1-D86-90R or different amounts of catalytically active BPLF1 and western blots were probed with the HA antibody. A band shift corresponding to monoubiquitinated TRIM25 was detected in the cells even upon transfection of a small amount of wild type BPLF1 but not in cells transfected with a 12-fold higher amount of the BPLF1-D86-90R mutant. (TIF) [file ppat.1008146.s003.tif]

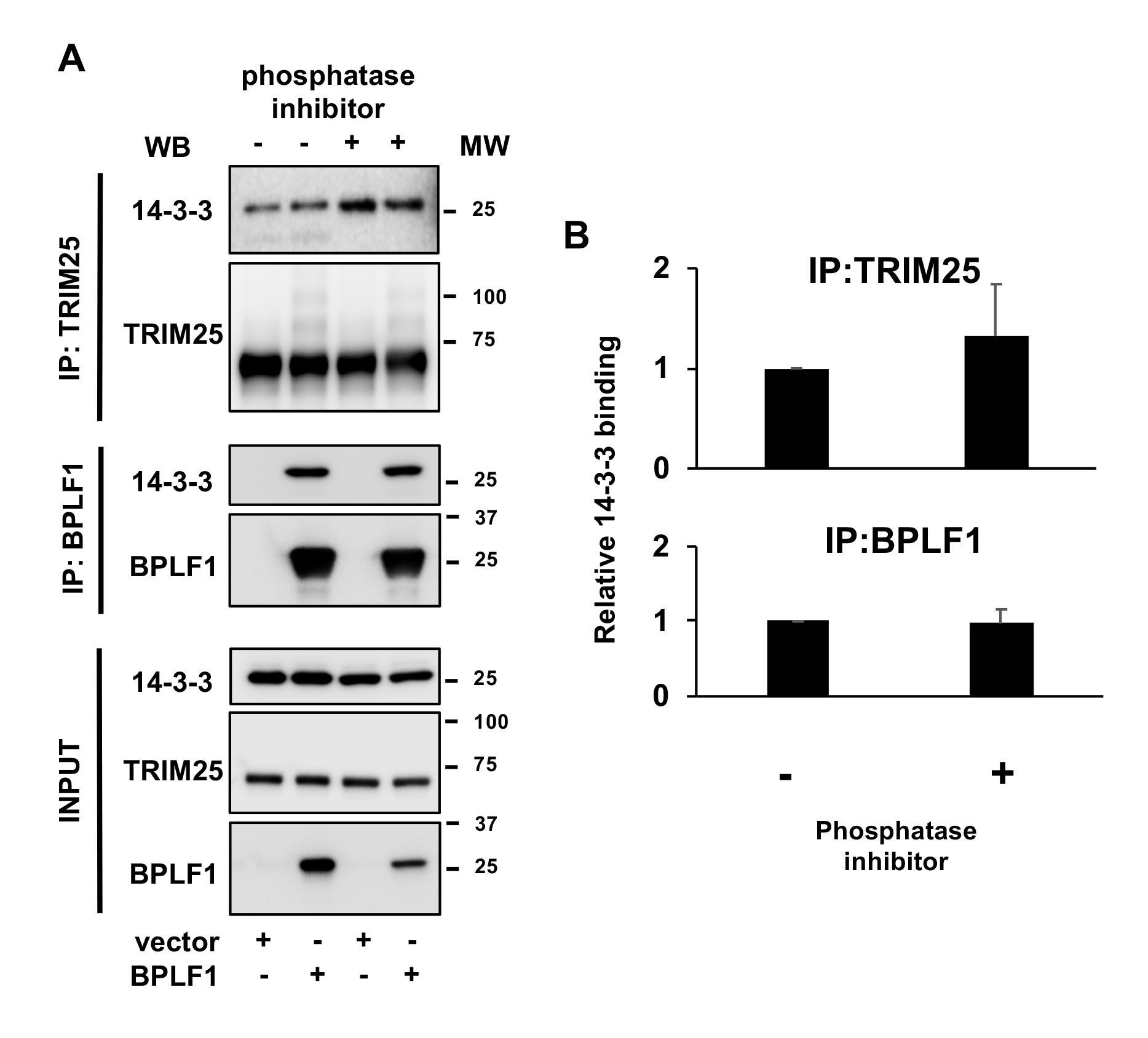

Supplement: S4 Fig — A. Cell lysates were prepared from Hela cells transfected with FLAG-BPLF1 in the presence or absence of phosphatase inhibitors. FLAG and TRIM25 immunoprecipitates were probed with antibodies to 14-3-3. One representative western blot out of three is shown in the figure. B. The intensity of the 14-3-3 specific bands were quantified by densitometry. Relative binding is expressed as the ratio of the intensity of the 14-3-3 band in the presence or absence of phosphatase inhibitors. The mean ± SD of three experiments is shown in the figure. (TIF) [file ppat.1008146.s004.tif]
